# Randomized Clinical Trials of Machine Learning Interventions in Health Care: A Systematic Review

**DOI:** 10.1001/jamanetworkopen.2022.33946

**Published:** 2022-09-29

**Authors:** Deborah Plana, Dennis L. Shung, Alyssa A. Grimshaw, Anurag Saraf, Joseph J. Y. Sung, Benjamin H. Kann

**Affiliations:** 1 Harvard Medical School, Boston, Massachusetts; 2 Department of Medicine, Yale University, New Haven, Connecticut; 3 Harvey Cushing/John Hay Whitney Medical Library, Yale University, New Haven, Connecticut; 4 Department of Radiation Oncology, Massachusetts General Hospital, Boston, Massachusetts; 5 Lee Kong Chian School of Medicine, Nanyang Technological University, Singapore; 6 Artificial Intelligence in Medicine Program, Brigham and Women’s Hospital, Harvard Medical School, Boston, Massachusetts

## Abstract

**Importance:**

Despite the potential of machine learning to improve multiple aspects of patient care, barriers to clinical adoption remain. Randomized clinical trials (RCTs) are often a prerequisite to large-scale clinical adoption of an intervention, and important questions remain regarding how machine learning interventions are being incorporated into clinical trials in health care.

**Objective:**

To systematically examine the design, reporting standards, risk of bias, and inclusivity of RCTs for medical machine learning interventions.

**Evidence Review:**

In this systematic review, the Cochrane Library, Google Scholar, Ovid Embase, Ovid MEDLINE, PubMed, Scopus, and Web of Science Core Collection online databases were searched and citation chasing was done to find relevant articles published from the inception of each database to October 15, 2021. Search terms for machine learning, clinical decision-making, and RCTs were used. Exclusion criteria included implementation of a non-RCT design, absence of original data, and evaluation of nonclinical interventions. Data were extracted from published articles. Trial characteristics, including primary intervention, demographics, adherence to the CONSORT-AI reporting guideline, and Cochrane risk of bias were analyzed.

**Findings:**

Literature search yielded 19 737 articles, of which 41 RCTs involved a median of 294 participants (range, 17-2488 participants). A total of 16 RCTS (39%) were published in 2021, 21 (51%) were conducted at single sites, and 15 (37%) involved endoscopy. No trials adhered to all CONSORT-AI standards. Common reasons for nonadherence were not assessing poor-quality or unavailable input data (38 trials [93%]), not analyzing performance errors (38 [93%]), and not including a statement regarding code or algorithm availability (37 [90%]). Overall risk of bias was high in 7 trials (17%). Of 11 trials (27%) that reported race and ethnicity data, the median proportion of participants from underrepresented minority groups was 21% (range, 0%-51%).

**Conclusions and Relevance:**

This systematic review found that despite the large number of medical machine learning–based algorithms in development, few RCTs for these technologies have been conducted. Among published RCTs, there was high variability in adherence to reporting standards and risk of bias and a lack of participants from underrepresented minority groups. These findings merit attention and should be considered in future RCT design and reporting.

## Key Points

**Question:**

How are machine learning interventions being incorporated into randomized clinical trials (RCTs) in health care?

**Findings:**

In this systematic review of 41 RCTs of machine learning interventions, despite the large number of medical machine learning–based algorithms in development, few RCTs for these technologies have been conducted. Of published RCTs, most did not fully adhere to accepted reporting guidelines and had limited inclusion of participants from underrepresented minority groups.

**Meaning:**

These findings highlight areas of concern regarding the quality of medical machine learning RCTs and suggest opportunities to improve reporting transparency and inclusivity, which should be considered in the design and publication of future trials.

## Teaser

This systematic review examines the design, reporting standards, risk of bias, and inclusivity of randomized clinical trials of machine learning interventions in health care.

## Introduction

Machine learning has the potential to improve the diagnosis and prognosis of disease to enhance clinical care. Given the increasing amount of digital data generated from routine medical care, available computational processing power, and research advances, such as deep learning, there has been substantial interest in applying machine learning techniques to improve patient care across medical disciplines.^1,2^ Models have been investigated for tasks such as improved cancer diagnosis, emergency department triage, and intensive care unit decision support.^3,4,5^ However, the recent failures to successfully implement machine learning systems in clinical settings have highlighted the limitations of this technology, generating disillusionment and distrust in their potential to impact medicine.^6,7^ These machine learning system failures are often attributable to a lack of generalizability, an inability to adapt a system trained with data from 1 context to perform well in a new one,^8^ or an inability to demonstrate a clinically meaningful benefit.^7^ Mitigation strategies have been proposed to ensure their applicability, such as the use of larger and more diverse data sets and direct collaborations with clinical experts in model development.^9,10,11^ We investigated a different and complementary area of study of machine learning model-testing procedures, randomized clinical trials (RCTs), which may affect their ultimate use in heterogeneous clinical settings.

Randomized clinical trials are considered the gold standard for assessing an intervention’s impact in clinical care,^12^ and the current landscape of RCTs for machine learning in health care continues to evolve. Randomized clinical trials, particularly those with transparent and reproducible methods, are important for demonstrating the clinical utility of machine learning interventions given the inherent opacity and black box nature of these models and the difficulty in deciphering the mechanistic basis for model predictions.^13,14^ Furthermore, machine learning model performance in the clinical setting is dependent on the training data that was used during model development and may not generalize well to patient populations that are out of the training data’s distribution.^15^ Factors such as geographic location^16^ and racial, ethnic, and sex characteristics of model training data are often overlooked; thus, RCTs that are inclusive of a range of demographic backgrounds are crucial to avoiding known biases that can be propagated and deepened based on flawed training data.^17,18^

Therefore, we performed a systematic review to better understand the landscape of machine learning RCTs and trial qualities that affect reproducibility, inclusivity, generalizability, and successful implementation of artificial intelligence (AI) or machine learning interventions in clinical care. We focused the review on trials that used AI or machine learning as a clinical intervention, with patients allocated randomly to either a treatment arm with a therapeutic intervention based on machine learning or a standard of care arm.

## Methods

This systematic review used the Preferred Reporting Items for Systematic Reviews and Meta-analyses (PRISMA)^19^ and Synthesis Without Meta-analysis (SWiM)^20^ reporting guidelines. The protocol was registered a priori (CRD42021230810).

### Search Strategy and Selection Criteria

A systematic search of the literature was conducted by a medical librarian (A.A.G.) in Cochrane Library, Google Scholar, Ovid Embase, Ovid MEDLINE, PubMed, Scopus, and Web of Science Core Collection databases to find relevant articles published from the inception of each database to October 15, 2021, and final searches were performed in all databases on this date. The search was peer reviewed by a second medical librarian using the Peer Review of Electronic Search Strategies (PRESS) guideline.^21^ Databases were searched using a combination of controlled vocabulary and free text terms for AI, clinical decision-making, and RCTs. The search was not limited by language or year. Details of the full search strategies are given in eAppendix 1 in the Supplement. The citationchaser^22^ package for R software, version 4.0.3 (R Foundation for Statistical Computing) was used to search the reference lists of included studies and to retrieve articles that cited the included studies to find additional relevant studies not retrieved by the database search.

**Figure 1.**
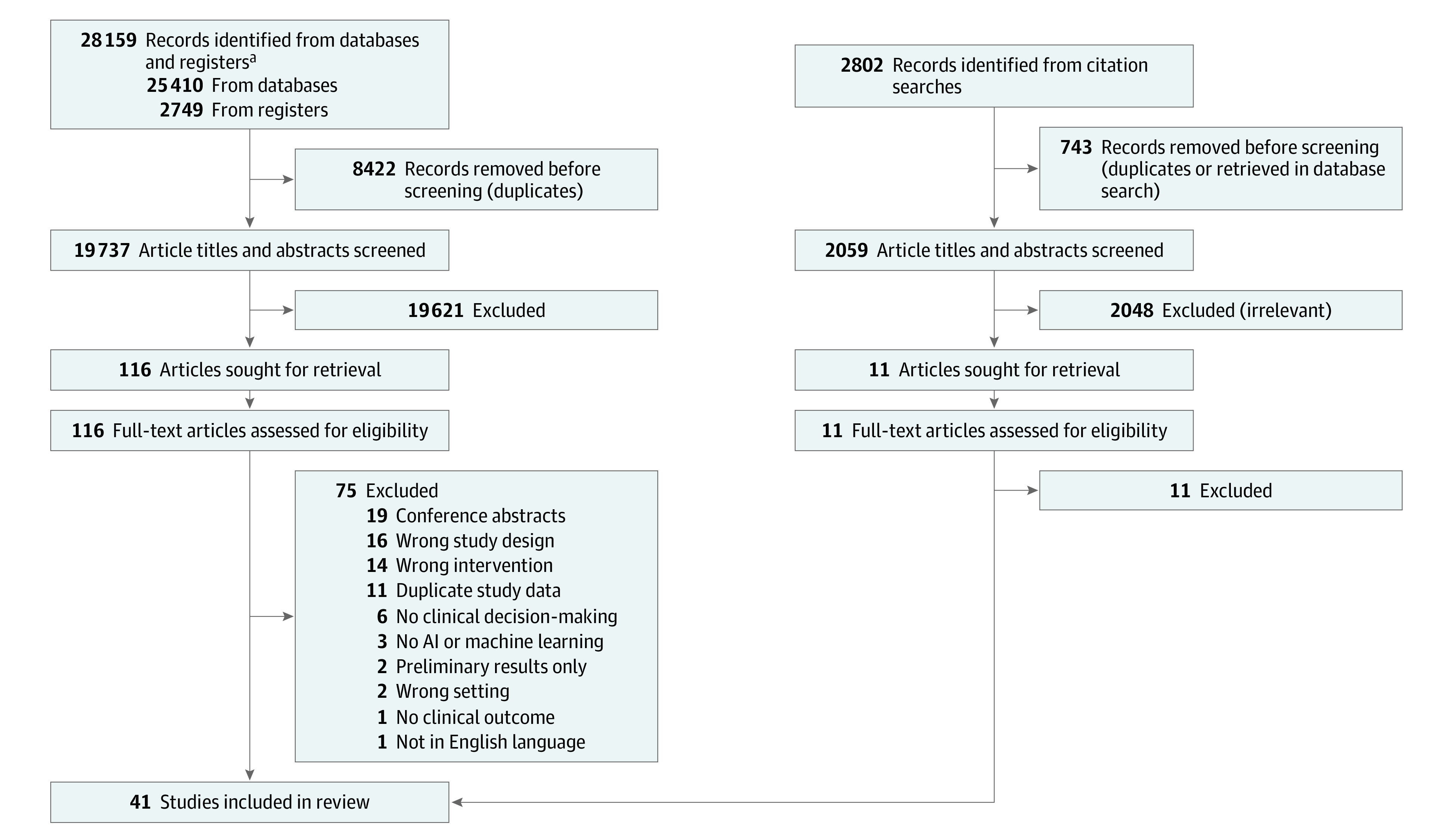
Screening and Selection of Randomized Clinical Trials AI indicates artificial intelligence. ^a^Databases and registers included Cochrane Library, Google Scholar, Ovid Embase, Ovid MEDLINE, PubMed, Scopus, and Web of Science Core Collection.

Citations from all databases were imported into an Endnote 20 library (Clarivate Analytics), in which duplicates were removed. The deduplicated results were imported into the Covidence systematic review management program for screening and data extraction. Two independent screeners performed a title and abstract review, with a third screener to resolve disagreements. This phase of screening was performed by 5 of us (D.P., D.L.S., A.A.G., A.S., and B.H.K.). The full texts of the resulting articles were then independently reviewed for inclusion by 2 screeners (D.P., D.L.S., and A.S.) and a third screener (B.H.K.) to resolve disagreements. Articles were included if they were deemed by consensus to be RCTs in which AI or machine learning was used in at least 1 randomization arm in a medical setting. Search strategies are available in the eAppendix 1 in the Supplement, and reasons for exclusion are found in Figure 1.

### Statistical Analysis

Two of us (D.P. and D.L.S.) independently extracted data and assessed the risk of bias for each study using standardized data extraction forms. The forms completed by each were compared; disagreement was resolved by review and discussion, with another one of us (B.H.K.) serving as final arbiter. Authors were not contacted for additional unpublished data. Risk of bias was assessed using the Cochrane Risk of Bias, version 2 tool for RCTs.^23^ This tool was developed to assess risk of bias in RCTs and has 5 domains, including risk of bias owing to the randomization process, deviations from the intended interventions (effect of assignment to intervention), missing outcome data, measurement of the outcome, and selection of the reported result.

To assess reproducibility and reporting transparency, we assessed article adherence to the recently published Consolidated Standards of Reporting Trials–Artificial Intelligence (CONSORT-AI) reporting guideline,^24^ which is an extension of the CONSORT guideline developed through an international multi-stakeholders group via staged consensus. Machine learning–based RCTs are recommended to routinely report the extended criteria in addition to the core CONSORT items. Two of us (D.P. and D.L.S.) independently extracted data and assessed each of the 11 CONSORT-AI extension criteria for each article. Disagreement was resolved by review and discussion, with another of us (B.H.K.) serving as final arbiter.

To evaluate inclusivity, we assessed reporting of sex, race, and ethnicity. We calculated the proportion of participants from underrepresented minority groups within each study using the National Institutes of Health definition of groups underrepresented in biomedical research^25^; the definition designates American Indian or Alaska Native, Black or African American, Hispanic or Latino, and Native Hawaiian or other Pacific Islander as underrepresented minority groups. To assess other qualities pertaining to generalizability and clinical adoption, we assessed the use of clinical vs nonclinical end points, whether the trial was conducted at a single site or multiple sites, and geographic location. Other qualities assessed were the use of measures with vs without performance thresholds, the disease setting of the trial, the model training data type, and the machine learning model type. The data for all aforementioned items were independently extracted by 2 of us (D.P. and D.L.S.) for each article, with disagreement resolved by review and discussion, with another of us (B.H.K.) serving as final arbiter. All summary statistics were computed using R software, version 4.0.3.

## Results

The search resulted in 28 159 records; after duplicates were removed, 19 737 remained for title and abstract screening, and 19 621 of these were excluded (Figure 1). No additional articles were located from citation chasing. Full-text review was conducted for 116 articles; of those, 75 studies were excluded because they were conference abstracts (n = 19), had the wrong study design (n = 16), performed the wrong intervention (n = 14), contained duplicate study data (n = 11), did not involve clinical decision-making (n = 6), did not use AI or machine learning (n = 3), provided preliminary results only (n = 2), were not conducted in a medical setting (n = 2), did not assess any outcomes that impacted clinical decision-making (n = 1), or were not written in the English language (n = 1) (eAppendix 1 and eAppendix 2 in the Supplement). Overall, 41 RCTs involving a median of 294 participants (range, 17-2488 participants) met inclusion criteria.^26,27,28,29,30,31,32,33,34,35,36,37,38,39,40,41,42,43,44,45,46,47,48,49,50,51,52,53,54,55,56,57,58,59,60,61,62,63,64,65,66^

**Figure 2.**
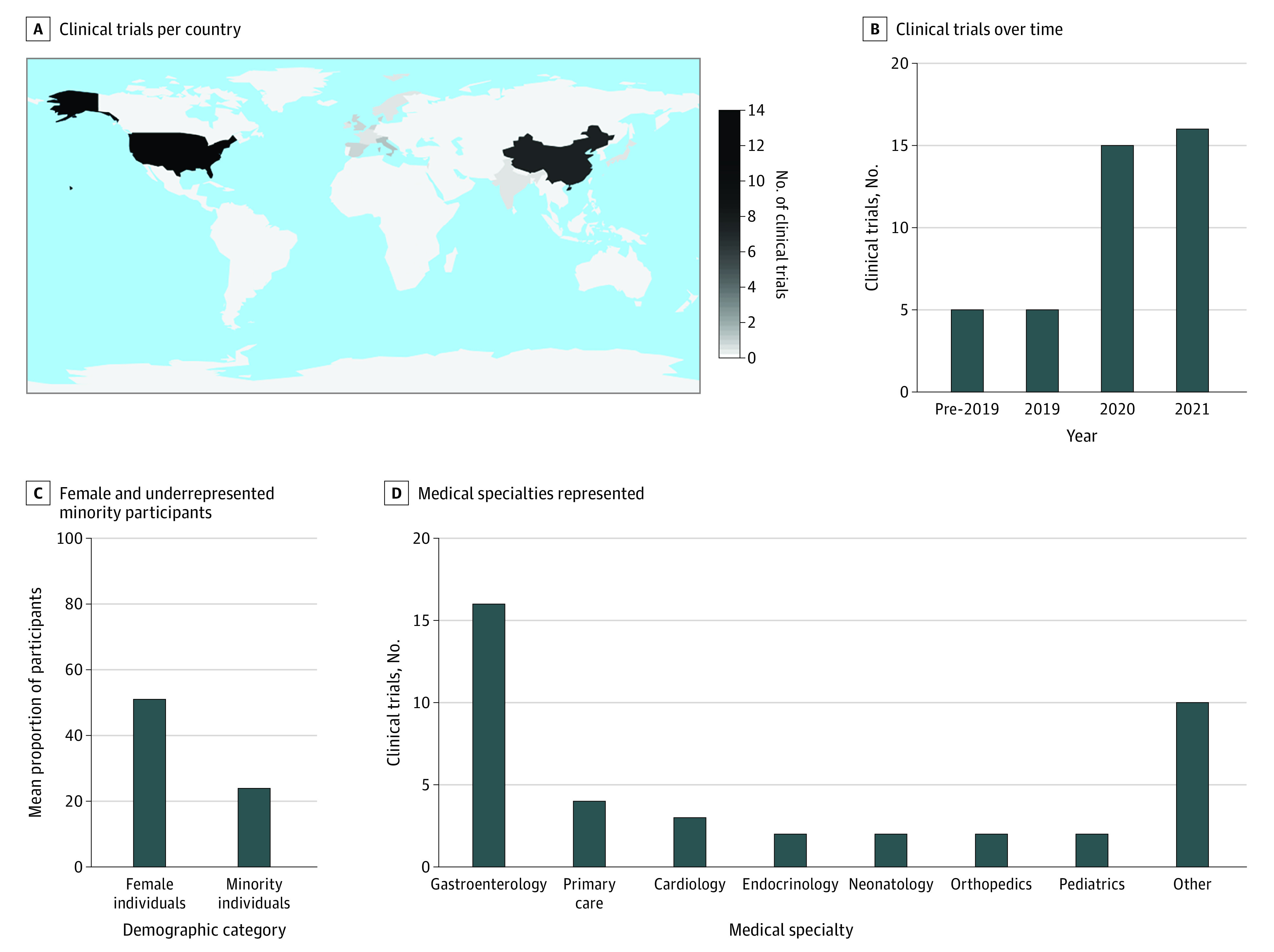
Characteristics of Randomized Clinical Trials A total of 41 randomized clinical trials were included in the analysis.^26,27,28,29,30,31,32,33,34,35,36,37,38,39,40,41,42,43,44,45,46,47,48,49,50,51,52,53,54,55,56,57,58,59,60,61,62,63,64,65,66^ Individuals from underrepresented minority groups were participants in 11 clinical trials in which information on participant race and/or ethnicity was reported.^30,31,32,33,36,44,49,55,59,61,63^ B, Data for 2021 are from January through October 15. D, The *other* medical specialty category includes anesthesiology, cardiac surgery, emergency medicine, general surgery, gynecology, intensive care, ophthalmology, pulmonology, and radiology.

The main study characteristics are shown in the Table as well as eAppendix 3 in the Supplement. No quantitative meta-analysis was performed owing to the heterogeneity of reported outcomes across clinical trials. The number of published RCTs increased substantially over the study period. Of the 41 included RCTs,^26,27,28,29,30,31,32,33,34,35,36,37,38,39,40,41,42,43,44,45,46,47,48,49,50,51,52,53,54,55,56,57,58,59,60,61,62,63,64,65,66^ 16 (39%) were published from January to October 2021^42,47,48,49,50,51,53,54,55,56,57,59,60,61,62,63^ and 36 (88%) from January 2019 to October 2021 (Figure 2).^26,27,29,31,32,33,34,35,37,38,39,41,42,43,44,45,47,48,49,50,51,52,53,54,55,56,57,58,59,60,61,62,63,64,65,66^ Trials were most often conducted in the US (15 [37%])^29,30,31,32,33,36,40,44,46,49,55,59,61,62,63^ or China (13 [32%]),^27,37,38,39,43,45,48,52,54,56,60,65,66^ and 6 studies (15%) were conducted across multiple countries.^26,29,42,47,50,57^ In terms of qualities associated with generalizability, 20 RCTs^26,28,29,30,32,33,34,39,42,44,47,50,51,54,56,57,59,62,63,64^ (49%) were conducted at multiple sites, and 21 RCTs (51%) were conducted at a single site.^27,31,35,36,37,38,40,41,43,45,46,48,49,52,53,55,58,60,61,65,66^ Only 11 trials (27%) reported race and ethnicity (Figure 2)^30,31,32,33,36,44,49,55,59,61,63^; among those trials, a median of 21% (range, 0%-51%) of participants were from underrepresented minority groups.

**Table.** Summary of Randomized Clinical Trials Included in the Systematic Review

Source	Study location	Study aim	Female, %	Race and ethnicity (%)	Medical specialty	Disease
Pavel et al,^26^ 2020	Ireland, the Netherlands Sweden, UK	Detect neonatal seizures	40	NR	Neonatology	Neonatal seizures
Wang et al,^27^ 2020	China	Detect colorectal adenomas	52	NR	Gastroenterology	Colon polyp and adenoma
Caparros-Gonzalez et al,^28^ 2018	Spain	Assess premature infant physiological response	41	NR	Neonatology	None
Nimri et al,^29^ 2020	Europe (multiple countries), Israel, US	Optimize insulin dose	52	NR	Endocrinology	Type 1 diabetes
Vennalaganti et al,^30^ 2018	US	Detect Barrett esophagus–associated neoplasia	24	African American (2), White (95), other (3)	Gastroenterology	Barrett esophagus–associated neoplasia
Voss et al,^31^ 2019	US	Improve socialization in children with autism spectrum disorder	11	Black (3), East Asian (24), Hispanic (23), Middle Eastern (1), South Asian (8), White/European American (35), unknown (17)	Pediatrics	Autism spectrum disorders
Manz et al,^32^ 2020	US	Increase serious illness conversations among patients with cancer	54	Non-Hispanic Black (20), non-Hispanic White (70), other (10)	Oncology	End of life
Persell et al,^33^ 2020	US	Improve blood pressure control in outpatients with hypertension	61	Black (35), Hispanic (8), White (52), other (9)	Primary care	Hypertension
Repici et al,^34^ 2020	Italy	Detect colorectal adenomas	51	NR	Gastroenterology	Colorectal neoplasia
Wijnberge et al,^35^ 2020	Netherlands	Detect intraoperative hypotension	43	NR	Cardiac surgery	Intraoperative hypotension
Shimabukuro et al,^36^ 2017	US	Predict outcomes in patients with sepsis	54	African American (11), Asian (16), Hispanic (21), White (47), other (5)	Intensive care	Sepsis
Wang et al,^37^ 2020	China	Detect colorectal adenomas	49	NR	Gastroenterology	Colon polyp and adenoma
Gong et al,^38^ 2020	China	Detect colorectal adenomas	51	NR	Gastroenterology	Colorectal adenomas
Lin et al,^39^ 2019	China	Diagnose childhood cataracts	55	NR	Ophthalmology	Childhood cataracts
Rabbi et al,^40^ 2015	US	Facilitate weight loss through automated personalized feedback for physical activity and diet	47	NR	Primary care	None
Auloge et al,^41^ 2020	France	Assess feasibility of AI/AR tool for vertebroplasty	65	NR	Orthopedics	Vertebral fracture
Avari et al,^42^ 2021	Spain, UK	Decrease hypoglycemia episodes with personalized bolus advice for people with type 1 diabetes	52	NR	Endocrinology	Type 1 diabetes
Wang et al,^43^ 2019	China	Detect colorectal adenomas	52	NR	Gastroenterology	Colon polyp and adenoma
Forman et al,^44^ 2019	US	Facilitate weight loss by predicting and preventing dietary lapses	85	Hispanic or non-White (30), non-Hispanic White (70)	Primary care	Obesity
Wu et al,^45^ 2019	China	Reduce rate of blind spots during EGD	52	NR	Gastroenterology	No specific disease
El Solh et al,^46^ 2009	US	Predict optimal CPAP using neural network to reduce titration failure	57	NR	Pulmonology	Obstructive sleep apnea
Luštrek et al,^47^ 2021	Belgium, Italy	Assess self-management of congestive heart failure using app and wristband	NR	NR	Cardiology	Congestive heart failure
Chen and Gao,^48^ 2021	China	Assess AI-based echocardiography for diagnosis of acute heart failure	36	NR	Cardiology	Acute left heart failure
Seol et al,^49^ 2021	US	Assess management of childhood asthma	43	White (72)	Pediatrics	Asthma
Repici et al,^50^ 2022	Italy, Switzerland	Investigate colon adenoma detection of nonexpert endoscopists	50	NR	Gastroenterology	Colorectal cancer screening
Kamba et al,^51^ 2021	Japan	Decrease colon adenoma miss rate	23	NR	Gastroenterology	Colorectal cancer screening
Liu et al,^52^ 2020	China	Increase polyp and adenoma detection with CADe	53	NR	Gastroenterology	Colorectal cancer screening
Blomberg et al,^53^ 2021	Denmark	Assess emergency-dispatched recognition of cardiac arrest during call	36	NR	Emergency medicine	Dispatcher assessment
Xu et al,^54^ 2021	China	Assess polyp detection of AI-assisted colonoscopy	49	NR	Gastroenterology	Colorectal cancer screening
Jayakumar et al,^55^ 2021	US	Evaluate effect of AI-enabled patient decision aid on knee osteoarthritis management	64	Asian (12), Black or African American (17), Hispanic or Latino (34), White (36)	Orthopedics	Osteoarthritis
Wu et al,^56^ 2021	China	Identify blind spots in EGD	52	NR	Gastroenterology	Early gastric cancer
Sandal et al,^57^ 2021	Denmark, Norway	Improve quality of life in patients with lower back pain using app	55	NR	Primary care	Lower back pain
Noor et al,^58^ 2020	India	Identify follicles in patients with ovarian stimulation	100	NR	Gynecology	Infertility
Yao et al,^59^ 2021	US	Identify patients with low ejection fraction from ECG data	54	Asian (1), Black or African American (2), White (94), other (2), missing (0.5)	Cardiology	Heart failure
Wu et al,^60^ 2021	China	Identify gastric neoplasms on EGD	54	NR	Gastroenterology	Early gastric neoplasia
Strömblad et al,^61^ 2021	US	Predict surgical case durations	83	Asian (8), Black (8), White (77), other (4), unknown (4)	Surgery	Solid tumor surgical procedures for gynecological and colorectal cancers
Eng et al,^62^ 2021	US	Assess skeletal age	46	NR	Radiology	Skeletal development
Glissen Brown et al,^63^ 2022	US	Reduce adenoma miss rate using computer-aided polyp detection	45	African American (21), White (68)	Gastroenterology	Colorectal cancer screening
Meijer et al,^64^ 2020	Netherlands	Reduce pain after surgical procedures	56	NR	Anesthesiology	Postoperative pain
Liu et al,^65^ 2020	China	Improve detection rate of polyps and adenomas	46	NR	Gastroenterology	Colon polyp and adenoma
Su et al,^66^ 2020	China	Improve detection rate of polyps and adenomas	51	NR	Gastroenterology	Colon polyp and adenoma

Abbreviations: AI, artificial intelligence; AR, artificial reality; CADe, computer-aided detection; CPAP, continuous positive airway pressure; EGD, esophagogastroduodenoscopy; NR, not reported.

**Figure 3.**
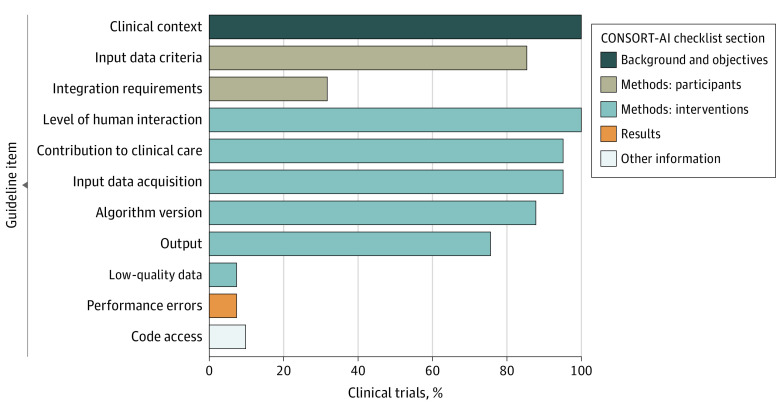
Adherence to Consolidated Standards of Reporting Trials–Artificial Intelligence (CONSORT-AI) Extension Guideline A total of 41 randomized clinical trials were included in the analysis.^26,27,28,29,30,31,32,33,34,35,36,37,38,39,40,41,42,43,44,45,46,47,48,49,50,51,52,53,54,55,56,57,58,59,60,61,62,63,64,65,66^ The CONSORT-AI extension is an internationally developed consensus document reflecting recommended clinical trial reporting characteristics to ensure transparency and reproducibility.^24^

To assess RCT transparency and reproducibility, we assessed trial adherence to CONSORT-AI standards (Figure 3). We found that no RCT met all the criteria. A total of 13 RCTs (32%) met at least 8 of 11 criteria (eAppendix 3 in the Supplement).^28,29,38,42,45,47,49,51,59,60,62,63,65^ The most common reasons for lack of guideline adherence were not assessing poor-quality or unavailable input data (38 trials [93%]),^26,27,28,29,30,31,32,33,34,35,36,37,39,40,41,42,43,44,45,46,48,49,50,52,53,54,55,56,57,58,59,60,61,62,63,64,65,66^ not analyzing performance errors (38 [93%]),^26,27,28,30,31,32,33,34,35,36,37,38,39,40,41,42,43,44,45,46,48,49,50,51,52,53,54,55,56,57,58,59,60,61,63,64,65,66^ and lack of a statement regarding code or algorithm availability (37 [90%]).^26,27,29,30,31,32,33,34,35,36,37,38,39,40,41,42,43,44,45,46,47,48,49,51,52,53,54,55,56,57,58,61,62,63,64,65,66^

**Figure 4.**
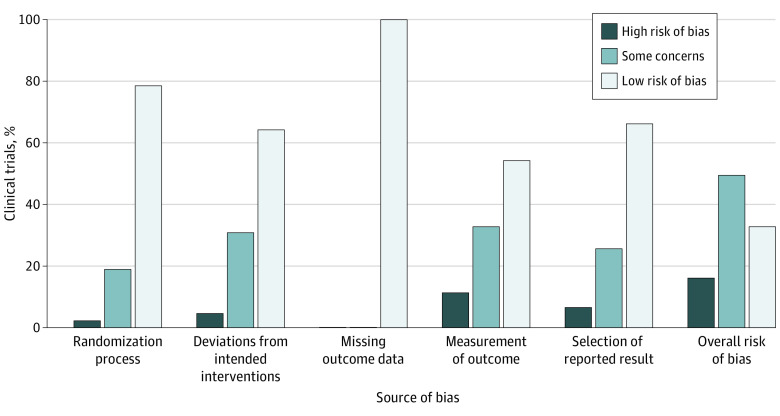
Risk of Bias in Randomized Clinical Trials A total of 41 randomized clinical trials were included in the analysis.^26,27,28,29,30,31,32,33,34,35,36,37,38,39,40,41,42,43,44,45,46,47,48,49,50,51,52,53,54,55,56,57,58,59,60,61,62,63,64,65,66^ Risk of bias was assessed using the revised Cochrane Risk of Bias, version 2 tool for randomized clinical trials.^23^

The risk of bias for the RCTs is summarized in Figure 4. Overall risk of bias was high in 7 trials (17%).^27,36,40,46,48,55,58^ Bias from measurement of outcomes was the type most often observed, with at least some concern for bias in 19 trials (46%).^27,33,38,40,41,42,43,44,45,46,48,49,51,55,56,59,63,65,66^

Regarding clinical use cases in RCTs, the most common clinical specialty represented was gastroenterology (16 [39%])^27,30,34,37,38,43,45,50,51,52,54,56,60,63,65,66^; most of these RCTs involved endoscopic imaging.^27,34,37,38,43,45,50,51,52,54,56,60,63,65,66^ Most studies involving clinical use cases enrolled adult patients (36 [88%]).^27,29,30,32,33,34,35,36,37,38,40,41,42,43,44,45,46,47,48,50,51,52,53,54,55,56,57,58,59,60,61,62,63,64,65,66^ Four trials (10%) were performed in a primary care setting, and all of these trials involved user-inputted data^33,40,44,57^; 4 other trials (10%) were in cardiology or cardiac surgery^35,47,48,59^ and involved electrocardiographic, wearable device, echocardiographic, or arterial waveform data. Two trials (5%) were performed in the neonatal setting,^26,28^ evaluating seizures and physiological distress, and 3 studies (7%) were performed primarily among pediatric populations more broadly,^31,39,49^ evaluating asthma, autism spectrum disorder, and childhood cataracts. Most RCTs involved clinical outcome measures (34 [83%])^26,27,29,30,31,32,33,34,35,36,37,38,39,42,43,44,45,46,48,49,50,51,52,53,54,55,57,58,59,60,63,64,65,66^ and outcome measures without performance thresholds (32 [78%]).^28,31,32,33,34,35,36,38,39,40,42,44,46,47,48,49,50,51,52,53,54,55,56,57,58,60,61,62,63,64,65,66^ In terms of data sources, 15 trials (37%) mostly used endoscopic imaging–based interventions,^27,34,37,38,43,45,50,51,52,54,56,60,63,65,66^ 5 (12%) used patient-inputted data,^33,40,44,55,57^ 2 (5%) used primary electronic health record data,^32,61^ 2 (5%) used electrocardiogram data,^48,59^ and 2 (5%) used blood-based data (glucose and insulin levels).^29,42^ A total of 20 articles (49%) used deep learning neural networks.^27,30,34,37,38,43,45,46,48,50,51,52,54,56,59,60,62,63,65,66^

## Discussion

This systematic review found a lack of RCTs for medical machine learning interventions and highlighted the need for additional well-designed, transparent, and inclusive RCTs for machine learning interventions to promote use in the clinic. There is growing concern that new machine learning models are being released after preliminary validation studies without follow-through on their ability to formally show superiority in a gold standard RCT.^67,68^ Of note, there are currently 343 US Food and Drug Administration (FDA)–approved medical AI or machine learning interventions.^69^ Our finding of 41 medical machine learning RCTs suggests that most FDA-approved machine learning–enabled medical devices are approved without efficacy demonstrated in an RCT. This finding is likely explained, in part, by the lower burden of evidence required for AI or machine learning algorithm clearance (often classified by the FDA as software as a medical device) compared with pharmaceutical drugs.^70^ To our knowledge, this review is the first rigorous attempt at quantifying this gap.

Prior work has shown a lack of prospective testing in this field but has not rigorously assessed the quantity of RCTs using a PROSPERO-registered method or tie-breaking arbitration^71^ or has analyzed the testing of technologies only related to imaging data.^72^ In addition, these studies did not explore study adherence to CONSORT-AI standards or assess the inclusivity of underrepresented minority groups and women in the study populations. Finally, the scope of our review compared with prior work differed; our work specifically focused on the use of clinical AI or machine learning interventions that were used as investigational arms in RCTs. We excluded RCTs that used traditional statistical models and RCTs in which AI or machine learning was included in the study protocol but was not part of the randomized intervention. In this way, we highlighted RCTs that directly compared AI or machine learning with standard of care and RCTs that were designed to demonstrate a high level of evidence for clinical utility. A comparison of the trials included in this study with prior work is available in eAppendix 4 in the Supplement.

Our initial search of 28 159 records and subsequent yield of only 41 RCTs^26,27,28,29,30,31,32,33,34,35,36,37,38,39,40,41,42,43,44,45,46,47,48,49,50,51,52,53,54,55,56,57,58,59,60,61,62,63,64,65,66^ indicates a translational gap between development and clinical impact. Most of the RCTs included in this review were conducted between January 2019 and October 2021 (36 [88%]),^26,27,29,31,32,33,34,35,37,38,39,41,42,43,44,45,47,48,49,50,51,52,53,54,55,56,57,58,59,60,61,62,63,64,65,66^ and 16 studies (39%) were conducted between January and October 2021,^42,47,48,49,50,51,53,54,55,56,57,59,60,61,62,63^ indicating that the rate of new RCTs for machine learning interventions increased over time. Clinical use cases for these technologies most often involved endoscopic imaging in gastroenterology (15 [37%])^27,34,37,38,43,45,50,51,52,54,56,60,63,65,66^ and enrolled adult patients (36 [88%]).^27,29,30,32,33,34,35,36,37,38,40,41,42,43,44,45,46,47,48,50,51,52,53,54,55,56,57,58,59,60,61,62,63,64,65,66^

Regarding trial reporting, no RCT included in this review adhered to all the machine learning-specific reporting standards (ie, the CONSORT-AI extension guideline^24^). Specifically, 37 trials (90%) did not share code and data along with study results,^26,27,29,30,31,32,33,34,35,36,37,38,39,40,41,42,43,44,45,46,47,48,49,51,52,53,54,55,56,57,58,61,62,63,64,65,66^ 38 (93%) did not analyze poor-quality or unavailable input data,^26,27,28,29,30,31,32,33,34,35,36,37,39,40,41,42,43,44,45,46,48,49,50,52,53,54,55,56,57,58,59,60,61,62,63,64,65,66^ and 38 (93%) did not assess performance errors,^26,27,28,30,31,32,33,34,35,36,37,38,39,40,41,42,43,44,45,46,48,49,50,51,52,53,54,55,56,57,58,59,60,61,63,64,65,66^ all of which may contribute to issues in reproducibility. These results suggest that machine learning RCT reporting quality needs improvement. The CONSORT-AI guideline helps ensure transparency and reproducibility of RCT methods,^24^ and the lack of guideline adherence observed among RCTs in this review may be another barrier to clinical adoption. Of note, the CONSORT-AI standards were published in September 2020, when most of the trials analyzed in this review would have been either published or under peer review. Future work should reassess the percentage of guideline-adherent RCTs published after 2021 to assess the impact of the CONSORT-AI guideline in RCT design.

Regarding RCT inclusivity, among the trials selected based on our search criteria, we found that only 20 (49%) were conducted at more than 1 site.^26,28,29,30,32,33,34,39,42,44,47,50,51,54,56,57,59,62,63,64^ In addition, we found a lack of reporting of demographic information across studies, with only 11 RCTs (27%) reporting participant race or ethnicity.^30,31,32,33,36,44,49,55,59,61,63^ Within this subset, studies had a median of 21% of enrolled participants belonging to underrepresented minority groups, a number concordant or slightly higher than proportions reported in prior systematic reviews analyzing medical RCTs.^73,74^ Trials were most often conducted in the US (15 [37%])^29,30,31,32,33,36,40,44,46,49,55,59,61,62,63^ or China (13 [32%]),^27,37,38,39,43,45,48,52,54,56,60,65,66^ and only 6 studies (15%) were conducted across multiple countries.^26,29,42,47,50,57^ Taken together, this lack of diversity in patient populations involved in RCTs indicates that the generalizability of their results across clinical sites is unknown—a growing concern for the federal regulation of machine learning interventions as medical devices.^75^

Regarding risk of bias, a high risk was found in 7 trials^27,36,40,46,48,55,58^ (17%); although substantial, this proportion was lower than the proportion of high-risk studies found in a cross-sectional study of non–machine learning interventions^76^ which found that a median of 50% of studies had a high risk of bias. This difference suggests that deficiencies in design, execution, and reporting of RCTs are not more widespread than those in other trials of medical interventions.

This systematic review found a low but increasing number of RCTs of machine learning interventions in health care. This low number is in contrast to the large number of preliminary validation studies of medical machine learning interventions and the increasing number of FDA approvals in this research area; many of these technologies have reached the clinical implementation phase without a gold standard assessment of efficacy through an RCT.^69^ It is not practical to formally assess every potential iteration of a new technology through an RCT (eg, a machine learning algorithm used in a hospital system and then used for the same clinical scenario in another geographic location). In particular, when an algorithm only indirectly affects patient care (eg, risk stratification, enhanced diagnosis), local, independent validation studies may provide an adequate level of evidence to encourage early adoption, although this is an area of ongoing debate. A baseline RCT of an intervention’s efficacy would help to establish whether a new tool provides clinical utility and value. This baseline assessment could be followed by retrospective or prospective external validation studies to demonstrate how an intervention’s efficacy generalizes over time and across clinical settings.

### Limitations

This study has several limitations. Of note, this analysis only selected RCTs that assessed a machine learning intervention directly impacting clinical decision-making. Additional work could be done to quantify the use of machine learning in alternative settings (eg, improving clinician-facing tools for workflow efficiency or assessing patient stratification, including biomarker discovery and validation efforts). Future work should aim to incorporate these wider definitions of a clinical tool for assessing the impact of machine learning across diverse steps in the clinical care pipeline. Nonetheless, we hypothesize that such literature contains a similar abundance of preliminary results and a dearth of RCTs assessing the relevance of machine learning in a controlled, clinical setting. An additional limitation is that this area of research is rapidly evolving, and our work is only current through October 2021. Future systematic reviews of machine learning interventions in health care will require more frequent updating and study of available results.

## Conclusions

This systematic review found a low but increasing number of RCTs for machine learning interventions in health care. These results highlight the need for medical machine learning RCTs to promote safe and effective clinical implementation. The findings also highlight areas of concern in terms of the quality of medical machine learning RCTs and opportunities to improve reporting transparency and inclusivity, which should be considered in the design and publication of future trials.

## Notes

Supplement. **eAppendix 1****.** Search Strategies **eAppendix 2.** Excluded Studies **eAppendix 3.** Included Trial Characteristics **eAppendix 4.** Comparison of Study Articles With Prior Literature
eReferences

## References

[1] Aung YYM, Wong DCS, Ting DSW. The promise of artificial intelligence: a review of the opportunities and challenges of artificial intelligence in healthcare. Br Med Bull. 2021;139(1):4-15. doi:10.1093/bmb/ldab016 34405854

[2] Wang F, Casalino LP, Khullar D. Deep learning in medicine—promise, progress, and challenges. JAMA Intern Med. 2019;179(3):293-294. doi:10.1001/jamainternmed.2018.7117 30556825

[3] Yue W, Wang Z, Chen H, Payne A, Liu X. Machine learning with applications in breast cancer diagnosis and prognosis. Designs. 2018;2(2):13. doi:10.3390/designs2020013

[4] Raita Y, Goto T, Faridi MK, Brown DFM, Camargo CA Jr, Hasegawa K. Emergency department triage prediction of clinical outcomes using machine learning models. Crit Care. 2019;23(1):64. doi:10.1186/s13054-019-2351-7 30795786 PMC6387562

[5] Johnson AEW, Ghassemi MM, Nemati S, Niehaus KE, Clifton DA, Clifford GD. Machine learning and decision support in critical care. Proc IEEE Inst Electr Electron Eng. 2016;104(2):444-466. doi:10.1109/JPROC.2015.2501978 27765959 PMC5066876

[6] Asan O, Bayrak AE, Choudhury A. Artificial intelligence and human trust in healthcare: focus on clinicians. J Med internet Res. 2020;22(6):e15154. doi:10.2196/15154 32558657 PMC7334754

[7] Wilkinson J, Arnold KF, Murray EJ, . Time to reality check the promises of machine learning–powered precision medicine. Lancet Digit Health. 2020;2(12):e677-e680. doi:10.1016/S2589-7500(20)30200-4 33328030 PMC9060421

[8] Zech JR, Badgeley MA, Liu M, Costa AB, Titano JJ, Oermann EK. Variable generalization performance of a deep learning model to detect pneumonia in chest radiographs: A cross-sectional study. PLoS Med. 2018;15(11):e1002683. doi:10.1371/journal.pmed.1002683 30399157 PMC6219764

[9] Vollmer S, Mateen BA, Bohner G, . Machine learning and artificial intelligence research for patient benefit: 20 critical questions on transparency, replicability, ethics, and effectiveness. BMJ. 2020;368:l6927. doi:10.1136/bmj.l6927 32198138 PMC11515850

[10] Davis SE, Lasko TA, Chen G, Siew ED, Matheny ME. Calibration drift in regression and machine learning models for acute kidney injury. J Am Med Inform Assoc. 2017;24(6):1052-1061. doi:10.1093/jamia/ocx030 28379439 PMC6080675

[11] Riley RD, Ensor J, Snell KIE, . External validation of clinical prediction models using big datasets from e-health records or IPD meta-analysis: opportunities and challenges. BMJ. 2016;353:i3140. doi:10.1136/bmj.i3140 27334381 PMC4916924

[12] Harbour R, Miller J. A new system for grading recommendations in evidence based guidelines. BMJ. 2001;323(7308):334-336. doi:10.1136/bmj.323.7308.334 11498496 PMC1120936

[13] Price WN. Big data and black-box medical algorithms. Sci Transl Med. 2018;10(471):eaao5333. doi:10.1126/scitranslmed.aao5333 30541791 PMC6345162

[14] The Lancet Respiratory Medicine. Opening the black box of machine learning. Lancet Respir Med. 2018;6(11):801. doi:10.1016/S2213-2600(18)30425-9 30343029

[15] Finlayson SG, Subbaswamy A, Singh K, . The clinician and dataset shift in artificial intelligence. N Engl J Med. 2021;385(3):283-286. doi:10.1056/NEJMc2104626 34260843 PMC8665481

[16] Kaushal A, Altman R, Langlotz C. Geographic distribution of US cohorts used to train deep learning algorithms. JAMA. 2020;324(12):1212-1213. doi:10.1001/jama.2020.12067 32960230 PMC7509620

[17] Mhasawade V, Zhao Y, Chunara R. Machine learning and algorithmic fairness in public and population health. Nat Mach Intell. 2021;3(8):659-666. doi:10.1038/s42256-021-00373-4

[18] Vokinger KN, Feuerriegel S, Kesselheim AS. Mitigating bias in machine learning for medicine. Commun Med (Lond). 2021;1:25. doi:10.1038/s43856-021-00028-w 34522916 PMC7611652

[19] Page MJ, Moher D, Bossuyt PM, . PRISMA 2020 explanation and elaboration: updated guidance and exemplars for reporting systematic reviews. BMJ. 2021;372(160):n160. doi:10.1136/bmj.n160 33781993 PMC8005925

[20] Campbell M, McKenzie JE, Sowden A, . Synthesis without meta-analysis (SWiM) in systematic reviews: reporting guideline. BMJ. 2020;368:l6890. doi:10.1136/bmj.l6890 31948937 PMC7190266

[21] McGowan J, Sampson M, Salzwedel DM, Cogo E, Foerster V, Lefebvre C. PRESS peer review of electronic search strategies: 2015 guideline statement. J Clin Epidemiol. 2016;75:40-46. doi:10.1016/j.jclinepi.2016.01.021 27005575

[22] Haddaway NR, Grainger MJ, Gray CT. Citationchaser: A tool for transparent and efficient forward and backward citation chasing in systematic searching. Res Synth Methods. 2022;13(4):533-545. doi:10.1002/jrsm.1563 35472127

[23] Sterne JAC, Savović J, Page MJ, . RoB 2: a revised tool for assessing risk of bias in randomised trials. BMJ. 2019;366:l4898. doi:10.1136/bmj.l4898 31462531

[24] Liu X, Cruz Rivera S, Moher D, Calvert MJ, Denniston AK; SPIRIT-AI and CONSORT-AI Working Group. Reporting guidelines for clinical trial reports for interventions involving artificial intelligence: the CONSORT-AI extension. Nat Med. 2020;26(9):1364-1374. doi:10.1038/s41591-020-1034-x 32908283 PMC7598943

[25] Notice of NIH’s interest in diversity. News release. National Institutes of Health. November 22, 2019. Accessed February 23, 2022. https://grants.nih.gov/grants/guide/notice-files/NOT-OD-20-031.html

[26] Pavel AM, Rennie JM, de Vries LS, . A machine-learning algorithm for neonatal seizure recognition: a multicentre, randomised, controlled trial. Lancet Child Adolesc Health. 2020;4(10):740-749. doi:10.1016/S2352-4642(20)30239-X 32861271 PMC7492960

[27] Wang P, Liu P, Glissen Brown JR, . Lower adenoma miss rate of computer-aided detection-assisted colonoscopy vs routine white-light colonoscopy in a prospective tandem study. Gastroenterology. 2020;159(4):1252-1261. doi:10.1053/j.gastro.2020.06.023 32562721

[28] Caparros-Gonzalez RA, de la Torre-Luque A, Diaz-Piedra C, Vico FJ, Buela-Casal G. Listening to relaxing music improves physiological responses in premature infants: a randomized controlled trial. Adv Neonatal Care. 2018;18(1):58-69. doi:10.1097/ANC.0000000000000448 29045255

[29] Nimri R, Battelino T, Laffel LM, ; NextDREAM Consortium. Insulin dose optimization using an automated artificial intelligence–based decision support system in youths with type 1 diabetes. Nat Med. 2020;26(9):1380-1384. doi:10.1038/s41591-020-1045-7 32908282

[30] Vennalaganti PR, Kaul V, Wang KK, . Increased detection of Barrett’s esophagus–associated neoplasia using wide-area trans-epithelial sampling: a multicenter, prospective, randomized trial. Gastrointest Endosc. 2018;87(2):348-355. doi:10.1016/j.gie.2017.07.039 28757316

[31] Voss C, Schwartz J, Daniels J, . Effect of wearable digital intervention for improving socialization in children with autism spectrum disorder: a randomized clinical trial. JAMA Pediatr. 2019;173(5):446-454. doi:10.1001/jamapediatrics.2019.0285 30907929 PMC6503634

[32] Manz CR, Parikh RB, Small DS, . Effect of integrating machine learning mortality estimates with behavioral nudges to clinicians on serious illness conversations among patients with cancer: a stepped-wedge cluster randomized clinical trial. JAMA Oncol. 2020;6(12):e204759. doi:10.1001/jamaoncol.2020.4759 33057696 PMC7563672

[33] Persell SD, Peprah YA, Lipiszko D, . Effect of home blood pressure monitoring via a smartphone hypertension coaching application or tracking application on adults with uncontrolled hypertension: a randomized clinical trial. JAMA Netw Open. 2020;3(3):e200255. doi:10.1001/jamanetworkopen.2020.0255 32119093 PMC7052730

[34] Repici A, Badalamenti M, Maselli R, . Efficacy of real-time computer-aided detection of colorectal neoplasia in a randomized trial. Gastroenterology. 2020;159(2):512-520. doi:10.1053/j.gastro.2020.04.062 32371116

[35] Wijnberge M, Geerts BF, Hol L, . Effect of a machine learning–derived early warning system for intraoperative hypotension vs standard care on depth and duration of intraoperative hypotension during elective noncardiac surgery: the HYPE randomized clinical trial. JAMA. 2020;323(11):1052-1060. doi:10.1001/jama.2020.0592 32065827 PMC7078808

[36] Shimabukuro DW, Barton CW, Feldman MD, Mataraso SJ, Das R. Effect of a machine learning–based severe sepsis prediction algorithm on patient survival and hospital length of stay: a randomised clinical trial. BMJ Open Respir Res. 2017;4(1):e000234. doi:10.1136/bmjresp-2017-000234 29435343 PMC5687546

[37] Wang P, Liu X, Berzin TM, . Effect of a deep-learning computer-aided detection system on adenoma detection during colonoscopy (CADE-DB trial): a double-blind randomised study. Lancet Gastroenterol Hepatol. 2020;5(4):343-351. doi:10.1016/S2468-1253(19)30411-X 31981517

[38] Gong D, Wu L, Zhang J, . Detection of colorectal adenomas with a real-time computer-aided system (ENDOANGEL): a randomised controlled study. Lancet Gastroenterol Hepatol. 2020;5(4):352-361. doi:10.1016/S2468-1253(19)30413-3 31981518

[39] Lin H, Li R, Liu Z, . Diagnostic efficacy and therapeutic decision-making capacity of an artificial intelligence platform for childhood cataracts in eye clinics: a multicentre randomized controlled trial. EClinicalMedicine. 2019;9:52-59. doi:10.1016/j.eclinm.2019.03.001 31143882 PMC6510889

[40] Rabbi M, Pfammatter A, Zhang M, Spring B, Choudhury T. Automated personalized feedback for physical activity and dietary behavior change with mobile phones: a randomized controlled trial on adults. JMIR Mhealth Uhealth. 2015;3(2):e42. doi:10.2196/mhealth.4160 25977197 PMC4812832

[41] Auloge P, Cazzato RL, Ramamurthy N, . Augmented reality and artificial intelligence–based navigation during percutaneous vertebroplasty: a pilot randomised clinical trial. Eur Spine J. 2020;29(7):1580-1589. doi:10.1007/s00586-019-06054-6 31270676

[42] Avari P, Leal Y, Herrero P, . Safety and feasibility of the PEPPER adaptive bolus advisor and safety system: a randomized control study. Diabetes Technol Ther. 2021;23(3):175-186. doi:10.1089/dia.2020.0301 33048581

[43] Wang P, Berzin TM, Glissen Brown JR, . Real-time automatic detection system increases colonoscopic polyp and adenoma detection rates: a prospective randomised controlled study. Gut. 2019;68(10):1813-1819. doi:10.1136/gutjnl-2018-317500 30814121 PMC6839720

[44] Forman EM, Goldstein SP, Crochiere RJ, . Randomized controlled trial of OnTrack, a just-in-time adaptive intervention designed to enhance weight loss. Transl Behav Med. 2019;9(6):989-1001. doi:10.1093/tbm/ibz137 31602471

[45] Wu L, Zhang J, Zhou W, . Randomised controlled trial of WISENSE, a real-time quality improving system for monitoring blind spots during esophagogastroduodenoscopy. Gut. 2019;68(12):2161-2169. doi:10.1136/gutjnl-2018-317366 30858305 PMC6872441

[46] El Solh A, Akinnusi M, Patel A, Bhat A, TenBrock R. Predicting optimal CPAP by neural network reduces titration failure: a randomized study. Sleep Breath. 2009;13(4):325-330. doi:10.1007/s11325-009-0247-5 19259717

[47] Luštrek M, Bohanec M, Cavero Barca C, . A personal health system for self-management of congestive heart failure (HeartMan): development, technical evaluation, and proof-of-concept randomized controlled trial. JMIR Med Inform. 2021;9(3):e24501. doi:10.2196/24501 33666562 PMC7980114

[48] Chen J, Gao Y. The role of deep learning–based echocardiography in the diagnosis and evaluation of the effects of routine anti–heart-failure Western medicines in elderly patients with acute left heart failure. *J Healthc Eng*. 2021:4845792. doi:10.1155/2021/4845792 PMC8371608 34422243

[49] Seol HY, Shrestha P, Muth JF, . Artificial intelligence-assisted clinical decision support for childhood asthma management: A randomized clinical trial. PLoS One. 2021;16(8):e0255261. doi:10.1371/journal.pone.0255261 34339438 PMC8328289

[50] Repici A, Spadaccini M, Antonelli G, . Artificial intelligence and colonoscopy experience: lessons from two randomised trials. Gut. 2022;71(4):757-765. doi:10.1136/gutjnl-2021-324471 34187845

[51] Kamba S, Tamai N, Saitoh I, . Reducing adenoma miss rate of colonoscopy assisted by artificial intelligence: a multicenter randomized controlled trial. J Gastroenterol. 2021;56(8):746-757. doi:10.1007/s00535-021-01808-w 34218329

[52] Liu P, Wang P, Glissen Brown JR, . The single-monitor trial: an embedded CADe system increased adenoma detection during colonoscopy: a prospective randomized study. Therap Adv Gastroenterol. 2020;13:1756284820979165. doi:10.1177/1756284820979165 33403003 PMC7745558

[53] Blomberg SN, Christensen HC, Lippert F, . Effect of machine learning on dispatcher recognition of out-of-hospital cardiac arrest during calls to emergency medical services: a randomized clinical trial. JAMA Netw Open. 2021;4(1):e2032320. doi:10.1001/jamanetworkopen.2020.32320 33404620 PMC7788469

[54] Xu L, He X, Zhou J, . Artificial intelligence–assisted colonoscopy: a prospective, multicenter, randomized controlled trial of polyp detection. Cancer Med. 2021;10(20):7184-7193. doi:10.1002/cam4.4261 34477306 PMC8525182

[55] Jayakumar P, Moore MG, Furlough KA, . Comparison of an artificial intelligence–enabled patient decision aid vs educational material on decision quality, shared decision-making, patient experience, and functional outcomes in adults with knee osteoarthritis: a randomized clinical trial. JAMA Netw Open. 2021;4(2):e2037107. doi:10.1001/jamanetworkopen.2020.37107 33599773 PMC7893500

[56] Wu L, He X, Liu M, . Evaluation of the effects of an artificial intelligence system on endoscopy quality and preliminary testing of its performance in detecting early gastric cancer: a randomized controlled trial. Endoscopy. 2021;53(12):1199-1207. doi:10.1055/a-1350-5583 33429441

[57] Sandal LF, Bach K, Øverås CK, . Effectiveness of app-delivered, tailored self-management support for adults with lower back pain–related disability: a selfBACK randomized clinical trial. JAMA Intern Med. 2021;181(10):1288-1296. doi:10.1001/jamainternmed.2021.4097 34338710 PMC8329791

[58] Noor N, Vignarajan CP, Malhotra N, Vanamail P. Three-dimensional automated volume calculation (sonography-based automated volume count) versus two-dimensional manual ultrasonography for follicular tracking and oocyte retrieval in women undergoing in vitro fertilization-embryo transfer: a randomized controlled trial. J Hum Reprod Sci. 2020;13(4):296-302. doi:10.4103/jhrs.JHRS_91_20 33627979 PMC7879837

[59] Yao X, Rushlow DR, Inselman JW, . Artificial intelligence-enabled electrocardiograms for identification of patients with low ejection fraction: a pragmatic, randomized clinical trial. Nat Med. 2021;27(5):815-819. doi:10.1038/s41591-021-01335-4 33958795

[60] Wu L, Shang R, Sharma P, . Effect of a deep learning–based system on the miss rate of gastric neoplasms during upper gastrointestinal endoscopy: a single-centre, tandem, randomised controlled trial. Lancet Gastroenterol Hepatol. 2021;6(9):700-708. doi:10.1016/S2468-1253(21)00216-8 34297944

[61] Strömblad CT, Baxter-King RG, Meisami A, . Effect of a predictive model on planned surgical duration accuracy, patient wait time, and use of presurgical resources: a randomized clinical trial. JAMA Surg. 2021;156(4):315-321. doi:10.1001/jamasurg.2020.6361 33502448 PMC7841577

[62] Eng DK, Khandwala NB, Long J, . Artificial intelligence algorithm improves radiologist performance in skeletal age assessment: a prospective multicenter randomized controlled trial. Radiology. 2021;301(3):692-699. doi:10.1148/radiol.2021204021 34581608

[63] Glissen Brown JR, Mansour NM, Wang P, . Deep learning computer-aided polyp detection reduces adenoma miss rate: a United States multi-center randomized tandem colonoscopy study (CADeT-CS Trial). Clin Gastroenterol Hepatol. 2022;20(7):1499-1507. doi:10.1016/j.cgh.2021.09.009 34530161

[64] Meijer F, Honing M, Roor T, . Reduced postoperative pain using nociception level–guided fentanyl dosing during sevoflurane anaesthesia: a randomised controlled trial. Br J Anaesth. 2020;125(6):1070-1078. doi:10.1016/j.bja.2020.07.057 32950246 PMC7771114

[65] Liu WN, Zhang YY, Bian XQ, . Study on detection rate of polyps and adenomas in artificial-intelligence–aided colonoscopy. Saudi J Gastroenterol. 2020;26(1):13-19. doi:10.4103/sjg.SJG_377_19 31898644 PMC7045775

[66] Su JR, Li Z, Shao XJ, . Impact of a real-time automatic quality control system on colorectal polyp and adenoma detection: a prospective randomized controlled study (with videos). Gastrointest Endosc. 2020;91(2):415-424. doi:10.1016/j.gie.2019.08.026 31454493

[67] Kim DW, Jang HY, Kim KW, Shin Y, Park SH. Design characteristics of studies reporting the performance of artificial intelligence algorithms for diagnostic analysis of medical images: results from recently published papers. Korean J Radiol. 2019;20(3):405-410. doi:10.3348/kjr.2019.0025 30799571 PMC6389801

[68] Ben-Israel D, Jacobs WB, Casha S, . The impact of machine learning on patient care: a systematic review. Artif Intell Med. 2020;103:101785. doi:10.1016/j.artmed.2019.101785 32143792

[69] US Food and Drug Administration. Artificial intelligence and machine learning (AI/ML)–enabled medical devices. September 22, 2021. Accessed March 7, 2022. https://www.fda.gov/medical-devices/software-medical-device-samd/artificial-intelligence-and-machine-learning-aiml-enabled-medical-devices

[70] US Food and Drug Administration. Software as a medical device (SAMD). December 4, 2018. Accessed August 3, 2022. https://www.fda.gov/medical-devices/digital-health-center-excellence/software-medical-device-samd

[71] Zhou Q, Chen ZH, Cao YH, Peng S. Clinical impact and quality of randomized controlled trials involving interventions evaluating artificial intelligence prediction tools: a systematic review. NPJ Digit Med. 2021;4(1):154.doi:10.1038/s41746-021-00524-2 34711955 PMC8553754

[72] Nagendran M, Chen Y, Lovejoy CA, . Artificial intelligence versus clinicians: systematic review of design, reporting standards, and claims of deep learning studies. BMJ. 2020;368:m689. doi:10.1136/bmj.m689 32213531 PMC7190037

[73] Ma MA, Gutiérrez DE, Frausto JM, Al-Delaimy WK. Minority representation in clinical trials in the United States: trends over the past 25 years. Mayo Clin Proc. 2021;96(1):264-266. doi:10.1016/j.mayocp.2020.10.027 33413830

[74] Hoel AW, Kayssi A, Brahmanandam S, Belkin M, Conte MS, Nguyen LL. Under-representation of women and ethnic minorities in vascular surgery randomized controlled trials. J Vasc Surg. 2009;50(2):349-354. doi:10.1016/j.jvs.2009.01.012 19631869 PMC2759770

[75] US Food and Drug Administration. Artificial intelligence and machine learning in software as a medical device. September 22, 2021. Accessed February 23, 2022. https://www.fda.gov/medical-devices/software-medical-device-samd/artificial-intelligence-and-machine-learning-software-medical-device

[76] Hopewell S, Boutron I, Altman DG, Ravaud P. Incorporation of assessments of risk of bias of primary studies in systematic reviews of randomised trials: a cross-sectional study. BMJ Open. 2013;3(8):e003342. doi:10.1136/bmjopen-2013-003342 23975265 PMC3753473

