# Supplementary material for: Randomized Clinical Trials of Machine Learning Interventions in Health Care: A Systematic Review
Source: JAMA Netw Open. 2022 Sep 29;5(9):e2233946. doi: 10.1001/jamanetworkopen.2022.33946 (PMC9523495; doi:10.1001/jamanetworkopen.2022.33946)
Supplement: Supplement. — eAppendix 1. Search Strategies eAppendix 2. Excluded Studies eAppendix 3. Included Trial Characteristics eAppendix 4. Comparison of Study Articles With Prior Literature eReferences [file jamanetwopen-e2233946-s001.pdf]

Supplementary Online Content

Plana D, Shung DL, Grimshaw AA, Saraf A, Sung JJY, Kann BH. Randomized clinical trials of machine learning interventions in health care: a systematic review. *JAMA Netw Open*. 2022;5(9):e2233946. doi:10.1001/jamanetworkopen.2022.33946

eAppendix 1. Search Strategies

eAppendix 2. Excluded Studies

eAppendix 3. Included Trial Characteristics

eAppendix 4. Comparison of Study Articles With Prior Literature

eReferences

This supplementary material has been provided by the authors to give readers additional information about their work.

© 2022 Plana D et al. *JAMA Network Open*.

eAppendix 1: Search Strategies

Ovid Embase

1 exp artificial intelligence/
2 exp natural language processing/
3 deep learning/
4 ((artificial or computat* or computer* or machine or deep or transfer or hierarchical) adj1 (intelligence* or learning* or reasoning*)).tw,kw.
5 (neural network* or random forest* or decision tree* or knowledge representation* or computer vision system* or computer reasoning* or natural language processing or perceptron* or connectionist model* or expert system*).tw,kw.
6 AI.tw,kw.
7 or/1-6
8 exp diagnosis/
9 exp prediction/
10 exp clinical decision making/
11 exp computer assisted diagnosis/
12 (prognos* or predict* or diagnos*).tw,kw.
13 (decision making* or decision-making* or decision aid* or decision support* or clinical decision*).tw,kw.
14 diagnosis.fs.
15 8 or 9 or 10 or 11 or 12 or 13 or 14
16 7 and 15
17 limit 16 to (randomized controlled trial or controlled clinical trial)
18 exp clinical trial/
19 exp randomized controlled trial/
20 exp randomization/
21 exp single blind procedure/
22 exp double blind procedure/
23 exp crossover procedure/
24 exp placebo/
25 exp prospective study/
26 (randomi#ed controlled or rct or randomly allocated or allocated randomly or random allocation).ti,ab.
27 ((singl* or double or triple) adj1 (blind* or placebo*)).ti,ab.
28 (cross adj1 over).ti,ab.
29 or/18-28
30 16 and 29
31 exp animal/
32 exp animal/ and exp human/
33 31 not 32
34 30 not 33

Ovid MEDLINE(R) ALL

1 exp Artificial Intelligence/

© 2022 Plana D et al. *JAMA Network Open*.

2 ((artificial or computat* or computer* or machine or deep or transfer or hierarchical) adj1 (intelligence* or learning* or reasoning*)).tw,kf.
3 (neural network* or random forest* or decision tree* or knowledge representation* or computer vision system* or computer reasoning* or natural language processing* or perceptron* or connectionist model* or expert system*).tw,kf.
4 AI.tw,kf.
5 1 or 2 or 3 or 4
6 exp Diagnosis/
7 exp Clinical Decision-Making/
8 (prognos* or predict* or diagnos*).tw,kf.
9 (decision making* or decision-making* or decision aid* or decision support* or clinical decision*).tw,kf.
10 diagnosis.fs.
11 6 or 7 or 8 or 9 or 10
12 5 and 11
13 limit 12 to randomized controlled trial
14 exp clinical trial/
15 exp Randomized Controlled Trial/
16 Random Allocation/
17 single-blind method/
18 Double-Blind Method/
19 cross-over studies/
20 Prospective Studies/
21 (randomi#ed controlled or rct or randomly allocated or allocated randomly or random allocation).ti,ab.
22 ((singl* or double or triple) adj1 (blind* or placebo*)).ti,ab.
23 (cross adj1 over).ti,ab.
24 13 or 14 or 15 or 16 or 17 or 18 or 19 or 20 or 21 or 22 or 23
25 12 and 24
26 exp animals/
27 exp animals/ and exp humans/
28 26 not 27
29 25 not 28

Scopus

(TITLE-ABS-KEY ((artificial OR computat* OR computer* OR machine OR deep OR transfer OR hierarchical) W/1 (intelligence* OR learning* OR reasoning*)) OR TITLE-ABS-KEY ("neural network*" OR "random forest*" OR "decision tree*" OR "knowledge representation*" OR "computer vision system*" OR "computer reasoning*" OR "natural language processing*" OR perceptron* OR "connectionist model*" OR "expert system*")) AND (TITLE-ABS-KEY (prognos* OR predict* OR diagnos*) OR TITLE-ABS-KEY ("decision making*" OR "decision-making*" OR "decision aid*" OR "decision support*" OR "clinical decision*")) AND (TITLE-ABS-KEY (randomized OR randomised OR rct OR randomly AND allocated OR allocated AND randomly OR random AND allocation) OR TITLE-ABS-KEY ((singl* OR double OR triple) W/1 (blind* OR placebo*)) OR TITLE-ABS-KEY (cross W/1 over))

Web of Science Core Collection

© 2022 Plana D et al. *JAMA Network Open*.

#1 TS=((artificial or computat* or computer* or machine or deep or transfer or hierarchical) near/1 (intelligence* or learning* or reasoning*)) OR TS=("neural network*" or "random forest*" or "decision tree*" or "knowledge representation*" or "computer vision system*" or "computer reasoning*" or "natural language processing*" or perceptron* or "connectionist model*" or "expert system*")
#2 TS=(prognos* or predict* or diagnos*) OR TS=("decision making*" or "decision-making*" or "decision aid*" or "decision support*" or "clinical decision*")
#3 TS=(randomized or randomised or rct or randomly allocated or allocated randomly or random allocation) OR TS=((singl* or double or triple) near/1 (blind* or placebo*)) or TS=(cross near/1 over)
#4 #1 and #2 and #3

PubMed

(((randomized[Title/Abstract] OR randomised[Title/Abstract] OR rct[Title/Abstract] OR randomly allocated[Title/Abstract] OR allocated randomly[Title/Abstract] OR random allocation[Title/Abstract] OR cross over[Title/Abstract] OR placebo*[Title/Abstract] OR single blind*[Title/Abstract] OR double blind*[Title/Abstract] OR triple blind*[Title/Abstract]) AND (((("neural network*"[Title/Abstract] OR "random forest*"[Title/Abstract] OR "decision tree*"[Title/Abstract] OR "knowledge representation*"[Title/Abstract] OR "computer vision system*"[Title/Abstract] OR "computer reasoning*"[Title/Abstract] OR "natural language processing*"[Title/Abstract] OR perceptron*[Title/Abstract] OR "connectionist model*"[Title/Abstract] OR "expert system*")[Title/Abstract]) OR (artificial intelligence*[Title/Abstract] OR computat* intelligence*[Title/Abstract] OR computer* intelligence*[Title/Abstract] OR machine intelligence*[Title/Abstract] OR deep intelligence*[Title/Abstract] OR transfer intelligence*[Title/Abstract] OR hierarchical intelligence*[Title/Abstract] OR artificial learning*[Title/Abstract] OR computat* learning*[Title/Abstract] OR computer* learning*[Title/Abstract] OR machine learning*[Title/Abstract] OR deep learning*[Title/Abstract] OR transfer learning*[Title/Abstract] OR hierarchical learning*[Title/Abstract] OR artificial reasoning*[Title/Abstract] OR computat* reasoning*[Title/Abstract] OR computer* reasoning*[Title/Abstract] OR machine reasoning*[Title/Abstract] OR deep reasoning*[Title/Abstract] OR transfer reasoning* or hierarchical reasoning*[Title/Abstract])) AND (prognos*[Title/Abstract] OR predict*[Title/Abstract] OR diagnos*[Title/Abstract] OR "decision making*"[Title/Abstract] OR "decision-making*"[Title/Abstract] OR "decision aid*"[Title/Abstract] OR "decision support*"[Title/Abstract] OR "clinical decision*"[Title/Abstract]))) OR (((("neural network*"[Title/Abstract] OR "random forest*"[Title/Abstract] OR "decision tree*"[Title/Abstract] OR "knowledge representation*"[Title/Abstract] OR "computer vision system*"[Title/Abstract] OR "computer reasoning*"[Title/Abstract] OR "natural language processing*"[Title/Abstract] OR perceptron*[Title/Abstract] OR "connectionist model*"[Title/Abstract] OR "expert system*")[Title/Abstract]) OR (artificial intelligence*[Title/Abstract] OR computat* intelligence*[Title/Abstract] OR computer* intelligence*[Title/Abstract] OR machine intelligence*[Title/Abstract] OR deep intelligence*[Title/Abstract] OR transfer intelligence*[Title/Abstract] OR hierarchical intelligence*[Title/Abstract] OR artificial learning*[Title/Abstract] OR computat* learning*[Title/Abstract] OR computer* learning*[Title/Abstract] OR machine learning*[Title/Abstract] OR deep learning*[Title/Abstract] OR transfer learning*[Title/Abstract] OR hierarchical learning*[Title/Abstract] OR artificial reasoning*[Title/Abstract] OR computat* reasoning*[Title/Abstract] OR computer*

reasoning*[Title/Abstract] OR machine reasoning*[Title/Abstract] OR deep reasoning*[Title/Abstract] OR transfer reasoning* or hierarchical reasoning*[Title/Abstract])) AND (prognos*[Title/Abstract] OR predict*[Title/Abstract] OR diagnos*[Title/Abstract] OR "decision making*"[Title/Abstract] OR "decision-making*"[Title/Abstract] OR "decision aid*"[Title/Abstract] OR "decision support*"[Title/Abstract] OR "clinical decision*"[Title/Abstract]) AND (randomizedcontrolledtrial[Filter]))

Cochrane Library

#1 ((artificial or computat* or computer* or machine or deep or transfer or hierarchical) near/1 (intelligence* or learning* or reasoning*)):ti,ab OR ("neural network*" or "random forest*" or "decision tree*" or "knowledge representation*" or "computer vision system*" or "computer reasoning*" or "natural language processing*" or perceptron* or "connectionist model*" or "expert system*"):ti,ab
#2 (prognos* or predict* or diagnos*):ti,ab OR ("decision making*" or "decision-making*" or "decision aid*" or "decision support*" or "clinical decision*"):ti,ab
#3 #1 and #2

Google Scholar

machine learning diagnosis randomized controlled trial

© 2022 Plana D et al. *JAMA Network Open*.

eAppendix 2: Excluded studies

First Authors Last Name	Year	Title	Journal	Reason for Exclusion
Abbas	2018	Machine learning approach for early detection of autism by combining questionnaire and home video screening	J Am Med Inform Assoc	Wrong study design
Auloge	2019	Augmented reality and artificial intelligence-based navigation during percutaneous vertebroplasty: A pilot prospective clinical study	CardioVascular and Interventional Radiology	Duplicate study data
Avari	2020	Efficacy and safety of the patient empowerment through predictive personalised decision support (PEPPER) system: an open-label randomised controlled trial	Diabetes Technology & Therapeutics	Duplicate study data
Avari	2020	Efficacy and safety of the patient empowerment through predictive personalised decision support (PEPPER) system: An open-label randomised controlled trial	Diabetes Technology and Therapeutics	Duplicate study data
Bai	2019	Establishment and real-world validation of a computer-assisted polyp identification and localization system based on deep learning	Journal of Digestive Diseases	Wrong study design
Barton	2018	Effect of a machine learning-based severe sepsis prediction algorithm on patient survival	Critical Care Medicine	No AI or machine learning
Ben-Yacov	2020	Personalized nutrition for prediabetes by prediction of glycemic responses	Diabetes. Conference: 80th Scientific Sessions of the American Diabetes Association, ADA	Conference abstract
Betancur	2018	Externally validated deep learning improves per-vessel prediction of obstructive coronary artery disease from upright and supine spect MPI: A multicenter study	Journal of Nuclear Cardiology	Wrong study design
Biester	2019	DREAM5: An open-label, randomized, cross-over study to evaluate the safety and efficacy of day and night closed-loop control by comparing the MD-Logic automated insulin delivery system to sensor augmented pump therapy in patients with type 1 diabetes at h	Diabetes, Obesity & Metabolism	Wrong intervention
Blomberg	2021	Effect of Machine Learning on Dispatcher Recognition of Out-of-Hospital Cardiac Arrest during Calls to Emergency Medical Services: A Randomized Clinical Trial	JAMA Network Open	Duplicate study data
Browning	2020	Results of the PReDicT Study: A Randomised Controlled Trial of Using the PReDicT Test to Guide Antidepressant Treatment in Depression	Biological Psychiatry	Conference abstract
Cai	2021	Improving Ki67 assessment concordance by the use of an artificial intelligence-empowered microscope: a multi-institutional ring study	Histopathology	Wrong study design
Chae	2020	Development and Clinical Evaluation of a Web-Based Upper Limb Home Rehabilitation System Using a Smartwatch and Machine Learning Model for Chronic Stroke Survivors: Prospective Comparative Study	JMIR Mhealth Uhealth	Wrong study design

© 2022 Plana D et al. *JAMA Network Open*.

Chiang	2021	Using Wearables and Machine Learning to Enable Personalized Lifestyle Recommendations to Improve Blood Pressure	Ieee Journal of Translational Engineering in Health and Medicine-Jtehm	Wrong study design
Chiang	2020	Physician Usage and Acceptance of a Machine Learning Recommender System for Simulated Clinical Order Entry	AMIA Summits on Translational Science Proceedings	No clinical decision making
Doupis	2018	Mobile-based artificial intelligence significantly improves type 1 diabetes management	Diabetes	Wrong study design
Dreiseitl	2009	Computer versus human diagnosis of melanoma: evaluation of the feasibility of an automated diagnostic system in a prospective clinical trial	Melanoma Research	Wrong study design
Elliott	2017	Clinical impact of pharmacogenetic profiling with a clinical decision support tool in polypharmacy home health patients: A prospective pilot randomized controlled trial	PLoS ONE	No AI or machine learning
Emura	2021	Id: 3526254 a Novel Deep Learning Model to Facilitate Complete Systematic Photodocumentation during Upper Gi Endoscopy	Gastrointestinal Endoscopy	Conference abstract
Gu	2021	Follow-up of atrial fibrillation recurrence after ablation with a BigThumb electrocardiogram monitor. [Chinese]	Academic Journal of Second Military Medical University	Foreign language
Harada	2021	Efficacy of artificial-intelligence-driven differential-diagnosis list on the diagnostic accuracy of physicians: An open-label randomized controlled study	International Journal of Environmental Research and Public Health	No clinical decision making
Ishiyama	2021	Impact of the clinical use of artificial intelligence-assisted neoplasia detection for colonoscopy: a large-scale prospective, propensity score-matched study (with video)	Gastrointestinal Endoscopy.	Wrong study design
Jaroszewski	2019	Randomized controlled trial of an online machine learning-driven risk assessment and intervention platform for increasing the use of crisis services	Journal of Consulting & Clinical Psychology	Wrong setting
Kamba	2021	Id: 3519580 a Multicentre Randomized Controlled Trial to Verify the Reducibility of Adenoma Miss Rate of Colonoscopy Assisted with Artificial Intelligence Based Software	Gastrointestinal Endoscopy	Conference abstract
Koons	2020	Combat medic testing of a novel monitoring capability for early detection of hemorrhage	The journal of trauma and acute care surgery	Wrong study design
Kumar	2020	OrderRex clinical user testing: a randomized trial of recommender system decision support on simulated cases	J Am Med Inform Assoc	Wrong intervention
Labovitz	2017	Using Artificial Intelligence to Reduce the Risk of Nonadherence in Patients on Anticoagulation Therapy	Stroke	Wrong intervention
Lam	2021	Artificial intelligence-assisted colonoscopy improves adenoma detection in screening colonoscopy. A large-scale multi-center randomized controlled study	Journal of Gastroenterology and Hepatology	Conference abstract

© 2022 Plana D et al. *JAMA Network Open*.

Lees	2003	Cluster-randomized, controlled trial of computer-based decision support for selecting long-term anti-thrombotic therapy after acute ischaemic stroke	Qjm-an International Journal of Medicine	Wrong intervention
Leng	2021	Assessing rectal cancer treatment response using coregistered endorectal photoacoustic and us imaging paired with deep learning	Radiology	Wrong study design
Liu	2018	Evaluating the impact of an integrated computer-based decision support with person-centered analytics for the management of hypertension: A randomized controlled trial	Journal of Hypertension	Wrong intervention
Liu	2020	Impact of Deep Learning-based Optimization Algorithm on Image Quality of Low-dose Coronary CT Angiography with Noise Reduction: A Prospective Study	Academic Radiology	Wrong study design
Lugtenberg	2015	Exposure to and experiences with a computerized decision support intervention in primary care: results from a process evaluation	BMC Fam Pract	Wrong intervention
Luna	2021	Artificial intelligence application versus physical therapist for squat evaluation: a randomized controlled trial	Scientific reports	No clinical decision making
McKee	2019	Randomized clinical trial of a just-in-time intervention based on wearable biosensors detecting smoking gestures	Alcoholism: Clinical and Experimental Research	Wrong intervention
Milluzzo	2021	Incremental yield of artificial intelligence in follow-up screening colonoscopies-aninterim analysis	Endoscopy	Conference abstract
Milluzzo	2021	Id: 3522041 Incremental Yield of Artificial Intelligence in Follow-up Screening Colonoscopies - an Interim Analysis	Gastrointestinal Endoscopy	Conference abstract
Mirelman	2009	Effects of training with a robot-virtual reality system compared with a robot alone on the gait of individuals after stroke	Stroke	Wrong intervention
Nieminen	2002	Prospective and randomised public-health trial on neural network-assisted screening for cervical cancer in Finland: results of the first year	Int J Cancer	No clinical decision making
Nieminen	2003	Prospective and randomised public-health trial on neural network-assisted screening for cervical cancer in Finland: Results of the first year	International Journal of Cancer	Preliminary results only
Noriega	2021	Screening Diabetic Retinopathy Using an Automated Retinal Image Analysis System in Independent and Assistive Use Cases in Mexico: Randomized Controlled Trial	JMIR Formative Research	Wrong study design
Park	2020	Validation of the effectiveness of a digital integrated healthcare platform utilizing an AI-based dietary management solution and a real-time continuous glucose monitoring system for diabetes management: a randomized controlled trial	BMC Medical Informatics & Decision Making	Preliminary results only
Pelle	2020	Effect of the dr. Bart application on healthcare use and clinical outcomes in people with osteoarthritis of the knee and/or hip in the Netherlands; a randomized controlled trial	Osteoarthritis & Cartilage	No AI or machine learning
Peyro-Saint-Paul	2019	Mobile application for adverse drug reaction reporting by patients with relapsing remitting multiple sclerosis (vigipsep study): a national randomized controlled trial	European Journal of Clinical Pharmacology	Wrong intervention

© 2022 Plana D et al. *JAMA Network Open*.

Raoux	2021	Novel ai-based solution for supporting prostate cancer diagnosis increases the efficiency and accuracy of reporting in clinical routine	Laboratory Investigation	Conference abstract
Rapoport	2018	Computer-Based Driving in Dementia Decision Tool With Mail Support: Cluster Randomized Controlled Trial	Journal of Medical Internet Research	Wrong intervention
Rein	2021	Personalized diets by prediction of glycemic responses improve glycemic control in subjects with newly diagnosed t2d	Diabetes. Conference: 81st Scientific Sessions of the American Diabetes Association, ADA	Conference abstract
Repici	2020	876 Real-Time Computer Aided Diagnosis for Detection of Colorectal Neoplasia at Colonoscopy	Gastrointestinal Endoscopy	Duplicate study data
Repici	2021	Efficacy of real-time computer-aided detection of colorectal neoplasia in a non-expertsetting: A randomized controlled trial	Endoscopy	Conference abstract
Repici	2021	Id: 3523387 Efficacy of Real-Time Computer Aided Detection of Colorectal Neoplasia in a Non-Expert Setting: A Randomized Controlled Trial	Gastrointestinal Endoscopy	Conference abstract
Ringel	2012	Accuracy of robot-assisted placement of lumbar and sacral pedicle screws: a prospective randomized comparison to conventional freehand screw implantation	Spine	Wrong intervention
Sandal	2019	An App-Delivered Self-Management Program for People With Low Back Pain: Protocol for the selfBACK Randomized Controlled Trial	JMIR Research Protocols	No original data
Saposnik	2020	Therapeutic decisions in ms care: An international study comparing clinical judgement vs. Information from artificial intelligence-based models	Multiple Sclerosis Journal	Conference abstract
Segal	2016	Controlling glucose levels by personalized nutrition tailored to the microbiome	Diabetes Technology and Therapeutics	Conference abstract
Seok	2021	A personalized 3d-printed model for obtaining informed consent process for thyroid surgery: A randomized clinical study using a deep learning approach with mesh-type 3d modeling	Journal of Personalized Medicine	No clinical decision making
Seol	2021	Artificial intelligence-assisted clinical decision support for childhood asthma management: A randomized clinical trial	PLoS ONE	Duplicate study data
Srivannaboon	1997	A prospective clinical trial comparing a topographically guided artificial intelligence software system versus clinical expertise for fitting normal and pathologic corneas with contact lenses	IOVS	Conference abstract
Sun	2014	A computer-aided diagnostic algorithm improves the accuracy of transesophageal echocardiography for left atrial thrombi: a single-center prospective study	Journal of ultrasound in medicine : official journal of the American Institute of Ultrasound in Medicine	Wrong study design
Symons	2020	Predicting alcohol dependence treatment outcomes: a prospective comparative study of clinical psychologists versus 'trained' machine learning models	Addiction (Abingdon, England)	Wrong study design
Tanaka	2012	Effect of a human-type communication robot on cognitive function in elderly women living alone	Med Sci Monit	Wrong intervention

© 2022 Plana D et al. *JAMA Network Open*.

Vennalaganti	2015	Increased detection of barrett's esophagus (BE)-associated Neoplasia using wide-area trans-epithelial sampling in conjunction with 4-quadrant biopsies: interim results from a multi-center, prospective, randomized trial	Gastroenterology.	Duplicate study data
Walter	2012	Effect of adding a diagnostic aid to best practice to manage suspicious pigmented lesions in primary care: randomised controlled trial	BMJ (Clinical research ed.)	Wrong intervention
Walter	2012	Effect of adding a diagnostic aid to best practice to manage suspicious pigmented lesions in primary care: randomised controlled trial	Bmj-British Medical Journal	Wrong intervention
Wang	2020	859 Computer-Aided-Detection Embedded Colonoscopy Versus Routine Colonoscopy: A Prospective, Randomized Tandem Trial	Gastroenterology	Duplicate study data
Wang	2019	Colonoscopy with embedded deep learning computer-aided detection system improves adenoma detection without increasing physician fatigue: a prospective randomized study	United European Gastroenterology Journal	Conference abstract
Wang	2018	Assistance of a real-time automatic colon polyp detection system increases polyp and adenoma detection during colonoscopy: A prospective randomized controlled study	United European Gastroenterology Journal	Conference abstract
Wang	2018	Automatic polyp detection during colonoscopy increases adenoma detection: An interim analysis of a prospective randomized control study	Gastrointestinal Endoscopy	Wrong setting
Wang	2019	Application of Artificial Intelligence-based Image Optimization for Computed Tomography Angiography of the Aorta With Low Tube Voltage and Reduced Contrast Medium Volume	J Thorac Imaging	No clinical outcome
Wismuller	2020	A Prospective Randomized Clinical Trial for Measuring Radiology Study Reporting Time on Artificial Intelligence-Based Detection of Intracranial Hemorrhage in Emergent Care Head CT	SPIE Medical Imaging Conference - Biomedical Applications in Molecular, Structural, and Functional Imaging	No clinical decision making
Wu	2021	Evaluating the Effects of An Artificial Intelligence System on Endoscopy Quality and Preliminarily Testing its Performance on Detecting Early Gastric Cancer: a Randomized Controlled Trial	Endoscopy	Duplicate study data
Yu	2019	378 Randomized Controlled Trial of Wisense, a Real-Time Quality Improving System for Monitoring Blind Spots during Esophagogastroduodenoscopy	Gastrointestinal Endoscopy	Duplicate study data
Yu	2019	Improved adenoma detection with ENDOANGEL: A randomized controlled trial	United European Gastroenterology Journal	Duplicate study data
Yu	2021	1136P A clinically applicable cervical cancer artificial intelligence screening system for accurate cytopathological diagnosis: A multicenter population-based study and randomized controlled trial	Annals of Oncology	Conference abstract
Zamora	2015	A clinical decision support system can improves the quality of lipid-lowering therapy in coronary patients	European heart journal.	Conference abstract
Zhou	2019	A real-time automatic deep learning polyp detection system increases polyp and adenoma detection during colonoscopy: a prospective double-blind randomized study	Gastroenterology	Conference abstract

© 2022 Plana D et al. *JAMA Network Open*.

eAppendix 3: Included trial characteristics

Study Aim	Primary Outcome	ML technology data type	ML technology model type	Primary outcome clinical	Primary outcome thresholded	Tool tested at multiple sites	Overall Risk of Bias
Detect neonatal seizures[36]	Diagnostic accuracy	EEG	Not Reported	Yes	Yes	Yes	Some concerns
Detect colorectal adenomas[37]	Adenoma miss rate	Imaging	Deep learning	Yes	Yes	No	High
Premature infant physiological response[38]	Respiratory rate, systolic and diastolic blood pressure, and heart rate	Music	Not Reported	No	No	Yes	Some concerns
Optimize insulin-dose[39]	Percentage of time glucose in target range	Glucose levels, insulin delivery history and meal consumption, as reported through the insulin pump's bolus calculator	Artificial intelligence	Yes	Yes	Yes	Low
Detect Barrett's esophagus–associated neoplasia[40]	Rate of detection of high-grade dysplasia/esophageal adenocarcinoma	Histology	Neural network analysis	Yes	Yes	Yes	Low
Improve Socialization in Children With Autism Spectrum Disorder[41]	Four socialization measures	3D images	Computer vision	Yes	No	No	Some concerns
Increase Serious Illness Conversations	Percentage of patient encounters with an SIC	Structured EHR data	Not Reported	Yes	No	Yes	Some concerns

© 2022 Plana D et al. *JAMA Network Open*.

Among Patients With Cancer[42]							
Improve control of blood pressure in outpatients with hypertension[43]	Systolic blood pressure at 6 months	Blood pressure, pulse, user responses to prompts	Artificial intelligence	Yes	No	Yes	Some concerns
Detect colorectal adenomas[44]	Adenoma detection rate	Imaging	CNN	Yes	No	Yes	Low
Detect Intraoperative Hypotension[45]	Time-weighted average of hypotension during surgery	Arterial waveform data	Not Reported	Yes	No	No	Low
Predict Outcomes of Patients with Sepsis[46]	Length of stay	Demographic and physiological information	Not Reported	Yes	No	No	High
Detect colorectal adenomas[47]	Adenoma detection rate	Imaging	Deep learning	Yes	Yes	No	Low
Detect colorectal adenomas[48]	Adenoma detection rate	Imaging	Deep learning	Yes	No	No	Some concerns
Diagnose childhood cataracts[49]	Diagnostic performance for childhood cataracts	Ocular images	Collaborative AI cloud platform	Yes	No	Yes	Low
Facilitate weight loss through automated personalized feedback for physical activity and diet[50]	Amount of walking	Physical activity and dietary intake data collected solely from a mobile phone	Multi-armed bandit	No	No	No	High
Feasibility of AI/AR tool for vertebroplasty[51]	Technical feasibility	Imaging	Not Reported	No	Yes	No	Some concerns
Decrease hypoglycemia episodes with personalized bolus advice for people with type 1 diabetes[52]	Percentage time in range	Insulin levels, physical activity before a meal, lifestyle information	Case-based reasoning	Yes	No	Yes	Some concerns
Detect colorectal adenomas[53]	Adenoma detection rates	Imaging	Deep learning	Yes	Yes	No	Some concerns

© 2022 Plana D et al. *JAMA Network Open*.

Facilitate weight loss by predicting and preventing dietary lapses[54]	Weight-loss and satisfaction	Patient-reported survey answers	Not Reported	Yes	No	Yes	Some concerns
Reduce rate of blind spots during EGD[55]	Blind Spot Rate	Video	CNN	Yes	Yes	No	Some concerns
Predict optimal CPAP by neural network to reduce titration failure[56]	Time to optimal CPAP pressure	Polysomnography	ANN	Yes	No	No	High
Self-management of Congestive Heart Failure using app and wristband[57]	HRQOL and self-reported improvement in self-care	Sensing device (wristband)	Not Reported	No	No	Yes	Some concerns
AI-based Echocardiogram for diagnosis of acute heart failure[58]	Rehospitalization rate	Echocardiogram	CNN	Yes	No	No	High
Management of childhood asthma[59]	Occurrence of asthma exacerbation in 1 year	Clinician alert	Not Reported	Yes	No	No	Some concerns
Colon adenoma detection of non-expert endoscopists[60]	Adenoma detection rate	Imaging	CNN	Yes	No	Yes	Low
Decrease colon adenoma miss rate[61]	Adenoma miss rate	Imaging	CNN	Yes	No	Yes	Some concerns
Increase polyp and adenoma detection with CADe[62]	Adenoma detection rate	Imaging	Deep-learning	Yes	No	No	Some concerns
Emergency Dispatched recognition of cardiac arrest during call[63]	Rate of dispatcher recognition of subsequently confirmed cardiac arrest	Dispatcher alert	Not Reported	Yes	No	No	Low

© 2022 Plana D et al. *JAMA Network Open*.

Polyp detection of AI-assisted colonoscopy[64]	Polyp detection rate	Imaging	Deep learning	Yes	No	Yes	Some concerns
AI-enabled patient decision aid on knee OA management[65]	Decision quality (Knee OA Decision Quality Instrument)	PROM scores	Not Reported	Yes	No	No	High
Identify blind spots in EGD[66]	Number of blind spots	Imaging	CNN	No	No	Yes	Some concerns
Improve quality of life in patients with lower back pain with app[67]	RMDQ Score	User input	Not Reported	Yes	No	Yes	Low
Identify follicles in patients with ovarian stimulation[68]	Number of oocytes retrieved	Sonography	Not Reported	Yes	No	No	High
Identify patients with low ejection fraction from EKG data[69]	Low EF	ECG waveforms	CNN	Yes	Yes	Yes	Some concerns
Identify gastric neoplasms on EGD[70]	Gastric neoplasia miss rate	Imaging	CNN	Yes	No	No	Low
Predict surgical case durations[71]	Accurate prediction of duration of each scheduled surgery by mean absolute error	EHR data, including patient data, surgeon-associated statistics, procedure groups, operational factors, and clinical note text	Random Forest	No	No	No	Low
Skeletal age assessment[72]	Mean absolute difference between skeletal age dictated into radiologists' signed report	Images	CNN	No	No	Yes	Low
Computer-aided Polyp Detection	Adenoma miss rate	Endoscopic videos	CNN	Yes	No	Yes	Some concerns

© 2022 Plana D et al. *JAMA Network Open*.

Reduces Adenoma Miss Rate[73]							
Reducing pain after surgery[74]	Postoperative pain assessed in the PACU	Autonomic clinical data	Not Reported	Yes	No	Yes	Low
Improving the detection rate of polyps and adenomas[75]	Detection rate of polyps/adenomas in colonoscopy	Imaging	CNN	Yes	No	No	Some concerns
Improving the detection rate of polyps and adenomas[76]	Adenoma detection rate	Imaging	CNN	Yes	No	No	Some concerns

© 2022 Plana D et al. *JAMA Network Open*.

Study Adherence to CONSORT-AI Guidelines

Ref #	Did the study meet the 11 new extension items in the CONSORT-AI extension reporting guidelines? (yes/no)	1) Explain the intended use of the AI intervention in the context of the clinical pathway, including its purpose and its intended users (for example, healthcare professionals, patients, public).	2) State the inclusion and exclusion criteria at the level of the input data.	3) Describe how the AI intervention was integrated into the trial setting, including any onsite or offsite requirements.	4) State which version of the AI algorithm was used.	5) Describe how the input data were acquired and selected for the AI intervention.	6) Describe how poor quality or unavailable input data were assessed and handled.	7) Specify whether there was human–AI interaction in the handling of the input data, and what level of expertise was required of users.	8) Specify the output of the AI intervention	9) Explain how the AI intervention's outputs contributed to decision-making or other elements of clinical practice.	10) Describe results of any analysis of performance errors and how errors were identified, where applicable. If no such analysis was planned or done, justify why not.	11) State whether and how the AI intervention and/or its code can be accessed, including any restrictions to access or re-use.
36	No	Yes	Yes	Yes	No	Yes	No	Yes	Yes	Yes	No	No
37	No	Yes	Yes	Yes	No	Yes	No	No	Yes	Yes	No	No
38	No	Yes	Yes	Yes	Yes	Yes	No	No	Yes	Yes	No	Yes
39	No	Yes	Yes	Yes	No	Yes	No	Yes	Yes	Yes	Yes	No
40	No	Yes	Yes	No	No	Yes	No	Yes	Yes	Yes	No	No
41	No	Yes	Yes	Yes	No	Yes	No	Yes	Yes	No	No	No
42	No	Yes	Yes	Yes	No	Yes	No	No	Yes	Yes	No	No
43	No	Yes	No	Yes	Yes	Yes	No	Yes	Yes	Yes	No	No
44	No	Yes	Yes	No	Yes	Yes	No	Yes	Yes	Yes	No	No
45	No	Yes	Yes	Yes	No	Yes	No	No	Yes	Yes	No	No
46	No	Yes	Yes	No	No	Yes	No	No	Yes	Yes	No	No
47	No	Yes	Yes	No	No	Yes	No	Yes	Yes	Yes	No	No
48	No	Yes	Yes	Yes	Yes	Yes	Yes	Yes	Yes	Yes	No	No

© 2022 Plana D et al. *JAMA Network Open*.

49	No	Yes	Yes	Yes	No	Yes	No	Yes	Yes	Yes	No	No
50	No	Yes	No	Yes	No	Yes	No	Yes	Yes	Yes	No	No
51	No	Yes	Yes	Yes	No	Yes	No	No	Yes	Yes	No	No
52	No	Yes	Yes	Yes	Yes	Yes	No	Yes	Yes	Yes	No	No
53	No	Yes	No	Yes	No	Yes	No	Yes	Yes	Yes	No	No
54	No	Yes	Yes	Yes	No	Yes	No	Yes	Yes	Yes	No	No
55	No	Yes	Yes	Yes	Yes	Yes	No	Yes	Yes	Yes	No	No
56	No	Yes	Yes	No	No	No	No	Yes	Yes	Yes	No	No
57	No	Yes	Yes	Yes	No	Yes	Yes	Yes	Yes	Yes	Yes	No
58	No	Yes	Yes	Yes	Yes	Yes	No	No	Yes	Yes	No	No
59	No	Yes	Yes	Yes	Yes	Yes	No	Yes	Yes	Yes	No	No
60	No	Yes	Yes	Yes	No	Yes	No	Yes	Yes	No	No	Yes
61	No	Yes	Yes	Yes	Yes	Yes	Yes	Yes	Yes	Yes	No	No
62	No	Yes	No	Yes	No	No	No	Yes	Yes	Yes	No	No
63	No	Yes	Yes	Yes	No	Yes	No	Yes	Yes	Yes	No	No
64	No	Yes	Yes	Yes	No	Yes	No	Yes	Yes	Yes	No	No
65	No	Yes	Yes	Yes	No	Yes	No	No	Yes	Yes	No	No
66	No	Yes	Yes	Yes	No	Yes	No	Yes	Yes	Yes	No	No
67	No	Yes	No	Yes	No	Yes	No	Yes	Yes	Yes	No	No
68	No	Yes	Yes	No	No	Yes	No	Yes	Yes	Yes	No	No
69	No	Yes	Yes	Yes	No	Yes	No	Yes	Yes	Yes	No	Yes
70	No	Yes	Yes	Yes	Yes	Yes	No	Yes	Yes	Yes	No	Yes
71	No	Yes	Yes	Yes	No	Yes	No	No	Yes	Yes	No	No
72	No	Yes	Yes	Yes	No	Yes	No	Yes	Yes	Yes	Yes	No
73	No	Yes	Yes	Yes	Yes	Yes	No	Yes	Yes	Yes	No	No
74	No	Yes	Yes	Yes	Yes	Yes	No	No	Yes	Yes	No	No
75	No	Yes	Yes	Yes	Yes	Yes	No	Yes	Yes	Yes	No	No
76	No	Yes	Yes	Yes	No	Yes	No	Yes	Yes	Yes	No	No

© 2022 Plana D et al. *JAMA Network Open*.

eAppendix 4: Study article comparison and contrast with articles included in Zhou, Q., Chen, Zh., Cao, Yh. et al. Clinical impact and quality of randomized controlled trials involving interventions evaluating artificial intelligence prediction tools: a systematic review. NPJ Digit. Med. 4, 154 (2021).

**Included in present study and Zhou et al (n=14)**	**Reason for Exclusion**
Blomberg SN, Christensen HC, Lippert F, Ersbøll AK, Torp-Petersen C, Sayre MR, et al. Effect of Machine Learning on Dispatcher Recognition of Out-of-Hospital Cardiac Arrest During Calls to Emergency Medical Services: A Randomized Clinical Trial. JAMA network open. 2021;4(1):e2032320	N/A
Gong D, Wu L, Zhang J, Mu G, Shen L, Liu J, et al. Detection of colorectal adenomas with a real-time computer-aided system (ENDOANGEL): a randomised controlled study. The Lancet Gastroenterology & Hepatology. 2020;5(4):352-61.	N/A
Lin H, Li R, Liu Z, Chen J, Yang Y, Chen H, et al. Diagnostic Efficacy and Therapeutic Decision-making Capacity of an Artificial Intelligence Platform for Childhood Cataracts in Eye Clinics: A Multicentre Randomized Controlled Trial. EClinicalMedicine. 2019;9:52-9.	N/A
Manz CR, Parikh RB, Small DS, Evans CN, Chivers C, Regli SH, et al. Effect of Integrating Machine Learning Mortality Estimates With Behavioral Nudges to Clinicians on Serious Illness Conversations Among Patients With Cancer: A Stepped-Wedge Cluster Randomized Clinical Trial. JAMA oncology. 2020;6(12):e204759.	N/A

© 2022 Plana D et al. *JAMA Network Open*.

Meijer F, Honing M, Roor T, Toet S, Calis P, Olofsen E, et al. Reduced postoperative pain using Nociception Level-guided fentanyl dosing during sevoflurane anaesthesia: a randomised controlled trial. British journal of anaesthesia. 2020;125(6):1070-8.	N/A
Pavel AM, Rennie JM, de Vries LS, Blennow M, Foran A, Shah DK, et al. A machine-learning algorithm for neonatal seizure recognition: a multicentre, randomised, controlled trial. The Lancet Child & adolescent health. 2020;4(10):740-9.	N/A
Liu WN, Zhang YY, Bian XQ, Wang LJ, Yang Q, Zhang XD, et al. Study on detection rate of polyps and adenomas in artificial intelligence-aided colonoscopy. Saudi J Gastroenterol. 2020;26(1):13-9.	N/A
Su JR, Li Z, Shao XJ, Ji CR, Ji R, Zhou RC, et al. Impact of a real-time automatic quality control system on colorectal polyp and Index Articles adenoma detection: a prospective randomized controlled study (with videos). Gastrointestinal endoscopy. 2019;91(2):415-24.e4.	N/A
Repici A, Badalamenti M, Maselli R, Correale L, Radaelli F, Rondonotti E, et al. Efficacy of Real-Time Computer-Aided Detection of Colorectal Neoplasia in a Randomized Trial. Gastroenterology. 2020.	N/A
Shimabukuro DW, Barton CW, Feldman MD, Mataraso SJ, Das R. Effect of a machine learning-based severe sepsis prediction algorithm on patient survival and hospital length of stay: a randomised clinical trial. BMJ Open Respiratory Research. 2017;4(1).	N/A
Wang P, Berzin TM, Glissen Brown JR, Bharadwaj S, Becq A, Xiao X, et al. Real-time automatic detection system increases colonoscopic polyp and adenoma detection rates: a prospective randomised controlled study. Gut. 2019;68(10):1813-9.	N/A
Wang P, Liu X, Berzin TM, Glissen Brown JR, Liu P, Zhou C, et al. Effect of a deep-learning computer-aided detection system on adenoma detection during colonoscopy (CADe-DB trial): a double-blind randomised study. The Lancet Gastroenterology & Hepatology. 2020;5(4):343-51.	N/A

© 2022 Plana D et al. *JAMA Network Open*.

Wijnberge M, Geerts BF, Hol L, Lemmers N, Mulder MP, Berge P, et al. Effect of a Machine Learning-Derived Early Warning System for Intraoperative Hypotension vs Standard Care on Depth and Duration of Intraoperative Hypotension During Elective Noncardiac Surgery: The HYPE Randomized Clinical Trial. Jama. 2020.	N/A
Wu L, Zhang J, Zhou W, An P, Shen L, Liu J, et al. Randomised controlled trial of WISENSE, a real-time quality improving system for monitoring blind spots during esophagogastroduodenoscopy. Gut. 2019;68(12):2161-9	N/A
**Excluded in present study via Title/Abstract Screening* (n=10)**<br>***Based on reviewer consensus**	
Bailey TC, Chen Y, Mao Y, Lu C, Hackmann G, Micek ST, et al. A trial of a real-time Alert for clinical deterioration in Patients hospitalized on general medical wards. Journal of Hospital Medicine. 2013;8(5):236-42.	Did not meet study criteria for machine learning algorithm
Brier ME, Gaweda AE, Dailey A, Aronoff GR, Jacobs AA. Randomized trial of model predictive control for improved anemia management. Clin J Am Soc Nephrol. 2010;5(5):814-20.	Did not meet study criteria for machine learning algorithm
Caballero-Ruiz E, García-Sáez G, Rigla M, Villaplana M, Pons B, Hernando ME. A web-based clinical decision support system for gestational diabetes: Automatic diet prescription and detection of insulin needs. International journal of medical informatics. 2017;102:35-49.	Not a clinical intervention
Chen D, Wu L, Li Y, Zhang J, Liu J, Huang L, et al. Comparing blind spots of unsedated ultrafine, sedated, and unsedated conventional gastroscopy with and without artificial intelligence: a prospective, single-blind, 3-parallel-group, randomized, single-center trial. Gastrointestinal endoscopy. 2019;91(2):332-9.e3.	Not a clinical intervention; use of AI not the primary intervention being compared (secondary outcome)
Geersing GJ, Hendriksen JMT, Zuithoff NPA, Roes KC, Oudega R, Takada T, et al. Effect of tailoring anticoagulant treatment duration by applying a recurrence risk prediction model in patients with venous thromboembolism compared to usual care: A randomized controlled trial. PLoS medicine. 2020;17(6):e1003142.	Did not meet study criteria for machine learning algorithm

© 2022 Plana D et al. *JAMA Network Open*.

Hong JC, Eclov NCW, Dalal NH, et al. System for High-Intensity Evaluation During Radiation Therapy (SHIELD-RT): A Prospective Randomized Study of Machine Learning-Directed Clinical Evaluations During Radiation and Chemoradiation. J Clin Oncol. 2020;38(31):3652-3661.	Machine learning intervention not part of randomization
Martin CM, Vogel C, Grady D, Zarabzadeh A, Hederman L, Kellett J, et al. Implementation of complex adaptive chronic care: the Patient Journey Record system (PaJR). Journal of Evaluation in Clinical Practice. 2012;18(6):1226-34	Machine learning intervention not part of randomization; wrong study design (Community-based cohort study)
Sheridan SL, Draeger LB, Pignone MP, Keyserling TC, Simpson RJ, Jr., Rimer B, et al. A randomized trial of an intervention to improve use and adherence to effective coronary heart disease prevention strategies. BMC health services research. 2011;11:331	Did not meet study criteria for machine learning algorithm
Wang SV, Rogers JR, Jin Y, et al. Stepped-wedge randomised trial to evaluate population health intervention designed to increase appropriate anticoagulation in patients with atrial fibrillation. *BMJ Qual Saf*. 2019;28(10):835-842.	Machine learning intervention not part of randomization; wrong study design
Zeevi D, Korem T, Zmora N, et al. Personalized Nutrition by Prediction of Glycemic Responses. *Cell*. 2015;163(5):1079-1094.	Not a clinical intervention
**Removed in citation chasing (n=2)**	
Brocklehurst P., Field D., Keith Greene, Ed Juszczak, Robert Keith, Sara Kenyon, et al. Computerised interpretation of fetal heart rate during labour (INFANT): a randomised controlled trial. Lancet. 2017;389(10080):1719-29.	Did not meet study criteria for machine learning algorithm
Kougias P, Tiwari V, Sharath SE, Garcia A, Pathak A, Chen M, et al. A Statistical Model-driven Surgical Case Scheduling System Improves Multiple Measures of Operative Suite Efficiency: Findings From a Single-center, Randomized Controlled Trial. Annals of surgery. 2019;270(6):1000-4.	Did not meet study criteria for machine learning algorithm
**Not identified from study search criteria (n=40)**	

© 2022 Plana D et al. *JAMA Network Open*.

Luo Y, Zhang Y, Liu M, Lai Y, Liu P, Wang Z, et al. Artificial Intelligence-Assisted Colonoscopy for Detection of Colon Polyps: a Prospective, Randomized Cohort Study. Journal of gastrointestinal surgery : official journal of the Society for Surgery of the Alimentary Tract. 2020.	Not a randomized control trial
Allegra A, Marino A, Volpes A, Coffaro F, Scaglione P, Gullo S, et al. A randomized controlled trial investigating the use of a predictive nomogram for the selection of the FSH starting dose in IVF/ICSI cycles. Reproductive biomedicine online. 2017;34(4):429-38	Not a clinical intervention
Clemons M, Bouganim N, Smith S, Mazzarello S, Vandermeer L, Segal R, et al. Risk Model-Guided Antiemetic Prophylaxis vs Physician's Choice in Patients Receiving Chemotherapy for Early-Stage Breast Cancer: A Randomized Clinical Trial. JAMA oncology. 2016;2(2):225-31	Not a clinical intervention
Finkelstein SM, Lindgren BR, Robiner W, Lindquist R, Hertz M, Carlin BP, et al. A randomized controlled trial comparing health and quality of life of lung transplant recipients following nurse and computer-based triage utilizing home spirometry monitoring. Telemedicine journal and e-health : the official journal of the American Telemedicine Association. 2013;19(12):897-903.	Not a clinical intervention
Fulmer R, Joerin A, Gentile B, Lakerink L, Rauws M. Using Psychological Artificial Intelligence (Tess) to Relieve Symptoms of Depression and Anxiety: Randomized Controlled Trial JMIR Ment Health 2018;5(4):e64	Not a clinical intervention
Gerendas BS, Waldstein SM, Simader C, et al. Three-dimensional automated choroidal volume assessment on standard spectral-domain optical coherence tomography and correlation with the level of diabetic macular edema. *Am J Ophthalmol*. 2014;158(5):1039-1048.	Not a clinical intervention

© 2022 Plana D et al. *JAMA Network Open*.

Guenancia C, Stamboul K, Hachet O, Yameogo V, Garnier F, Gudjoncik A, et al. Clinical effectiveness of the systematic use of the GRACE scoring system (in addition to clinical assessment) for ischaemic outcomes and bleeding complications in the management of NSTEMI compared with clinical assessment alone: a prospective study. Heart and vessels. 2016;31(6):897-906.	Did not meet study criteria for machine learning algorithm
Hill JC, Whitehurst DG, Lewis M, Bryan S, Dunn KM, Foster NE, et al. Comparison of stratified primary care management for low back pain with current best practice (STarT Back): a randomised controlled trial. Lancet. 2011;378(9802):1560-71.	Did not meet study criteria for machine learning algorithm
Hsu JC, Chen YF, Chung WS, Tan TH, Chen T, Chiang JY. Clinical verification of a clinical decision support system for ventilator weaning. Biomedical engineering online. 2013;12 Suppl 1(Suppl 1):S4	Not a clinical intervention
Kappen TH, Moons AH, Wolfswinkel Lv, Kalkman CJ, Vergouwe Y, van Klei WA. Impact of Risk Assessments on Prophylactic Antiemetic Prescription and the Incidence of Postoperative Nausea and Vomiting A Cluster-randomized Trial. Anesthesiology. 2014	Did not meet study criteria for machine learning algorithm
Lauffenburger JC, Lewey J, Jan S, Makanji S, Ferro CA, Krumme AA, et al. Effectiveness of Targeted Insulin-Adherence Interventions for Glycemic Control Using Predictive Analytics Among Patients With Type 2 Diabetes: A Randomized Clinical Trial. JAMA network open. 2019;2(3):e190657.	Machine learning intervention not part of randomization
Mazurek MO, Parker RA, Chan J, Kuhlthau K, Sohl K. Effectiveness of the Extension for Community Health Outcomes Model as Applied to Primary Care for Autism: A Partial Stepped-Wedge Randomized Clinical Trial. JAMA pediatrics. 2020;174(5):e196306	Did not meet study criteria for machine learning algorithm
Nieuwlaat R, Hubers LM, Spyropoulos AC, Eikelboom JW, Connolly BJ, Van Spall HG, et al. Randomised comparison of a simple warfarin dosing algorithm versus a computerised anticoagulation management system for control of warfarin maintenance therapy. Thrombosis and haemostasis. 2012;108(6):1228-35	Did not meet study criteria for machine learning algorithm

© 2022 Plana D et al. *JAMA Network Open*.

Nieuwlaat R, Eikelboom JW, Schulman S, van Spall HG, Schulze KM, Connolly BJ, et al. Cluster randomized controlled trial of a simple warfarin maintenance dosing algorithm versus usual care among primary care practices. Journal of thrombosis and thrombolysis. 2014;37(4):435-42	Did not meet study criteria for machine learning algorithm
Persell SD, Lloyd-Jones DM, Friesema EM, Cooper AJ, Baker DW. Electronic health record-based patient identification and individualized mailed outreach for primary cardiovascular disease prevention: a cluster randomized trial. Journal of general internal medicine. 2013;28(4):554-60.	Not a clinical intervention
Pielmeier U, Rousing ML, Andreassen S, Nielsen BS, Haure P. Decision support for optimized blood glucose control and nutrition in a neurotrauma intensive care unit: preliminary results of clinical advice and prediction accuracy of the Glucosafe system. Journal of clinical monitoring and computing. 2012;26(4):319-28	Did not meet study criteria for machine learning algorithm
Plomb-Holmes C, Hilfiker R, Leger B, Luthi F. Impact of a non-return-to-work prognostic model (WORRK) on allocation to rehabilitation clinical pathways: A single centre parallel group randomised trial. PloS one. 2018;13(8):e0201687.	Not a clinical intervention
Sadasivam RS, Borglund EM, Adams R, Marlin BM, Houston TK. Impact of a Collective Intelligence Tailored Messaging System on Smoking Cessation: The Perspect Randomized Experiment. Journal of medical Internet research. 2016;18(11).	Not a clinical intervention
Sherratt FC, Marcus MW, Robinson J, Field JK. Utilizing Lung Cancer Risk Prediction Models to Promote Smoking Cessation: Two Randomized Controlled Trials. American Journal of Health Promotion. 2016;32(5):1196-205.	Not a clinical intervention
Steiner JF, Shainline MR, Bishop MC, Xu S. Reducing Missed Primary Care Appointments in a Learning Health System. Medical care. 2016.	Not a clinical intervention
Steiner JF, Shainline MR, Dahlgren JZ, Kroll A, Xu S. Optimizing Number and Timing of Appointment Reminders: A Randomized Trial. Am J Manag Care. 2018.	Not a clinical intervention

© 2022 Plana D et al. *JAMA Network Open*.

True MW, Strickland LE, Lewi JE, Sterling LM, Dai H, Haas RW, et al. Impact of a Diabetes Risk Score on Lifestyle Education and Patient Adherence (IDEA) in Prediabetes: A Multisite Randomized Controlled Trial. Military medicine. 2015;180(10):1091-7.	Not a clinical intervention
Thurtle DR, Jenkins V, Pharoah PD, Gnanapragasam VJ. Understanding of prognosis in non-metastatic prostate cancer: a randomised comparative study of clinician estimates measured against the PREDICT prostate prognostic model. Br J Cancer. 2019;121(8):715-8	Not a clinical intervention
Cox CE, White DB, Hough CL, Jones DM, Kahn JM, Olsen MK, et al. Effects of a Personalized Web-Based Decision Aid for Surrogate Decision Makers of Patients With Prolonged Mechanical Ventilation. Ann Intern Med. 2019;170(5)	Did not meet study criteria for machine learning algorithm
de Vos-Kerkhof E, Nijman RG, Vergouwe Y, Polinder S, Steyerberg EW, van der Lei J, et al. Impact of a clinical decision model for febrile children at risk for serious bacterial infections at the emergency department: a randomized controlled trial. PloS one. 2015;10(5):e0127620.	Did not meet study criteria for machine learning algorithm
Mahler SA, Riley RF, Hiestand BC, Russell GB, Hoekstra JW, Lefebvre CW, et al. The HEART Pathway randomized trial: identifying emergency department patients with acute chest pain for early discharge. Circulation Cardiovascular quality and outcomes. 2015;8(2):195-203.	Did not meet study criteria for machine learning algorithm
Mán E, Simonka Z, Varga A, Rárosi F, Lázár G. Impact of the Alvarado score on the diagnosis of acute appendicitis: comparing clinical judgment, Alvarado score, and a new modified score in suspected appendicitis: a prospective, randomized clinical trial. Surgical endoscopy. 2014;28(8):2398-405.	Did not meet study criteria for machine learning algorithm
Mann D, Hess R, McGinn T, Richardson S, Jones S, Palmisano J, et al. Impact of Clinical Decision Support on Antibiotic Prescribing for Acute Respiratory Infections: a Cluster Randomized Implementation Trial. Journal of general internal medicine. 2020;35(Suppl 2):788-95.	Did not meet study criteria for machine learning algorithm

© 2022 Plana D et al. *JAMA Network Open*.

McGinn TG, McCullagh L, Kannry J, Knaus M, Sofianou A, Wisnivesky JP, et al. Efficacy of an evidence-based clinical decision support in primary care practices: a randomized clinical trial. JAMA internal medicine. 2013;173(17):1584-91	Did not meet study criteria for machine learning algorithm
Palen TE, Sharpe RE, Jr., Shetterly SM, Steiner JF. Randomized Clinical Trial of a Clinical Decision Support Tool for Improving the Appropriateness Scores for Ordering Imaging Studies in Primary and Specialty Care Ambulatory Clinics. AJR Am J Roentgenol. 2019;213(5):1015-20.	Did not meet study criteria for machine learning algorithm
Poldervaart JM, Reitsma JB, Backus BE, Koffijberg H, Veldkamp RF, Ten Haaf ME, et al. Effect of Using the HEART Score in Patients With Chest Pain in the Emergency Department: A Stepped-Wedge, Cluster Randomized Trial. Ann Intern Med. 2017;166(10):689-97	Did not meet study criteria for machine learning algorithm
Tan WJ, Acharyya S, Chew MH, Foo FJ, Chan WH, Wong WK, et al. Randomized control trial comparing an Alvarado Score-based management algorithm and current best practice in the evaluation of suspected appendicitis. World journal of emergency surgery : WJES. 2020;15(1):30	Did not meet study criteria for machine learning algorithm
Torres FA, Pasarelli I, Cutri A, Ossorio MF, Ferrero F. Impact assessment of a decision rule for using antibiotics in pneumonia: a randomized trial. Pediatric pulmonology. 2014;49(7):701-6.	Did not meet study criteria for machine learning algorithm
van de Maat JS, Peeters D, Nieboer D, van Wermeskerken AM, Smit FJ, Noordzij JG, et al. Evaluation of a clinical decision rule to guide antibiotic prescription in children with suspected lower respiratory tract infection in The Netherlands: A stepped-wedge cluster randomised trial. PLoS medicine. 2020;17(1):e1003034.	Did not meet study criteria for machine learning algorithm
Van Driest SL, Wang L, McLemore MF, Bridges BC, Fleming GM, McGregor TL, et al. Acute kidney injury risk-based screening in pediatric inpatients: a pragmatic randomized trial. Pediatric research. 2020;87(1):118-24	Did not meet study criteria for machine learning algorithm
K B, A. JH, O. AM. Quality improvement of functional diagnostics in dentistry through computer-aided diagnosis: a randomized controlled trial. International Journal of Computerized Dentistry. 2018	Machine learning intervention not part of randomization

© 2022 Plana D et al. *JAMA Network Open*.

Sáenz A, Brito M, Morón I, Torralba A, García-Sanz E, Redondo J. Development and validation of a computer application to aid the physician's decision-making process at the start of and during treatment with insulin in type 2 diabetes: a randomized and controlled trial. Journal of diabetes science and technology. 2012;6(3):581-8.	Did not meet study criteria for machine learning algorithm
Snooks H, Bailey-Jones K, Burge-Jones D, Dale J, Davies J, Evans B, et al. Predictive risk stratification model: a randomised steppedwedge trial in primary care (PRISMATIC). Health Services and Delivery Research. 2018;6(1):1-164.	Did not meet study criteria for machine learning algorithm
Stiell IG, Clement CM, Grimshaw JM, Brison RJ, Rowe BH, Lee JS, et al. A prospective cluster-randomized trial to implement the Canadian CT Head Rule in emergency departments. CMAJ : Canadian Medical Association journal = journal de l'Association medicale canadienne. 2010;182(14):1527-32.	Did not meet study criteria for machine learning algorithm
Steinhart BD, Levy P, Vandenberghe H, Moe G, Yan AT, Cohen A, et al. A Randomized Control Trial Using a Validated Prediction Model for Diagnosing Acute Heart Failure in Undifferentiated Dyspneic Emergency Department Patients-Results of the GASP4Ar Study. Journal of cardiac failure. 2016;23(2):145-52	Not a clinical intervention

© 2022 Plana D et al. *JAMA Network Open*.

eReferences

1. Aung YYM, Wong DCS, Ting DSW. The promise of artificial intelligence: a review of the opportunities and challenges of artificial intelligence in healthcare. *Br Med Bull*. 2021;139(1):4-15. doi:10.1093/bmb/ldab016

2. Wang F, Casalino LP, Khullar D. Deep Learning in Medicine—Promise, Progress, and Challenges. *JAMA Intern Med*. 2019;179(3):293. doi:10.1001/jamainternmed.2018.7117

3. Yue W, Wang Z, Chen H, Payne A, Liu X. Machine Learning with Applications in Breast Cancer Diagnosis and Prognosis. *Designs*. 2018;2(2):13. doi:10.3390/designs2020013

4. Raita Y, Goto T, Faridi MK, Brown DFM, Camargo CA, Hasegawa K. Emergency department triage prediction of clinical outcomes using machine learning models. *Crit Care*. 2019;23(1):64. doi:10.1186/s13054-019-2351-7

5. Johnson AEW, Ghassemi MM, Nemati S, Niehaus KE, Clifton D, Clifford GD. Machine Learning and Decision Support in Critical Care. *Proc IEEE*. 2016;104(2):444-466. doi:10.1109/JPROC.2015.2501978

6. Asan O, Bayrak AE, Choudhury A. Artificial Intelligence and Human Trust in Healthcare: Focus on Clinicians. *J Med Internet Res*. 2020;22(6):e15154. doi:10.2196/15154

7. Wilkinson J, Arnold KF, Murray EJ, et al. Time to reality check the promises of machine learning-powered precision medicine. *Lancet Digit Health*. 2020;2(12):e677-e680. doi:10.1016/S2589-7500(20)30200-4

8. Zech JR, Badgeley MA, Liu M, Costa AB, Titano JJ, Oermann EK. Variable generalization performance of a deep learning model to detect pneumonia in chest radiographs: A cross-sectional study. Sheikh A, ed. *PLOS Med*. 2018;15(11):e1002683. doi:10.1371/journal.pmed.1002683

9. Vollmer S, Mateen BA, Bohner G, et al. Machine learning and artificial intelligence research for patient benefit: 20 critical questions on transparency, replicability, ethics, and effectiveness. *BMJ*. Published online March 20, 2020:l6927. doi:10.1136/bmj.l6927

10. Davis SE, Lasko TA, Chen G, Siew ED, Matheny ME. Calibration drift in regression and machine learning models for acute kidney injury. *J Am Med Inform Assoc*. 2017;24(6):1052-1061. doi:10.1093/jamia/ocx030

11. Riley RD, Ensor J, Snell KIE, et al. External validation of clinical prediction models using big datasets from e-health records or IPD meta-analysis: opportunities and challenges. *BMJ*. Published online June 22, 2016:i3140. doi:10.1136/bmj.i3140

12. Harbour R, Miller J. A new system for grading recommendations in evidence based guidelines. *BMJ*. 2001;323(7308):334-336. doi:10.1136/bmj.323.7308.334

13. Price WN. Big data and black-box medical algorithms. *Sci Transl Med*. 2018;10(471):eaao5333. doi:10.1126/scitranslmed.aao5333

14. The Lancet Respiratory Medicine. Opening the black box of machine learning. *Lancet Respir Med*. 2018;6(11):801. doi:10.1016/S2213-2600(18)30425-9

15. Finlayson SG, Subbaswamy A, Singh K, et al. The Clinician and Dataset Shift in Artificial Intelligence. *N Engl J Med*. 2021;385(3):283-286. doi:10.1056/NEJMc2104626

16. Kaushal A, Altman R, Langlotz C. Geographic Distribution of US Cohorts Used to Train Deep Learning Algorithms. *JAMA*. 2020;324(12):1212. doi:10.1001/jama.2020.12067

© 2022 Plana D et al. *JAMA Network Open*.

17. Mhasawade V, Zhao Y, Chunara R. Machine learning and algorithmic fairness in public and population health. *Nat Mach Intell*. 2021;3(8):659-666. doi:10.1038/s42256-021-00373-4

18. Vokinger KN, Feuerriegel S, Kesselheim AS. Mitigating bias in machine learning for medicine. *Commun Med*. 2021;1(1):25. doi:10.1038/s43856-021-00028-w

19. Page MJ, Moher D, Bossuyt PM, et al. PRISMA 2020 explanation and elaboration: updated guidance and exemplars for reporting systematic reviews. *BMJ*. Published online March 29, 2021:n160. doi:10.1136/bmj.n160

20. Campbell M, McKenzie JE, Sowden A, et al. Synthesis without meta-analysis (SWiM) in systematic reviews: reporting guideline. *BMJ*. Published online January 16, 2020:l6890. doi:10.1136/bmj.l6890

21. McGowan J, Sampson M, Salzwedel DM, Cogo E, Foerster V, Lefebvre C. PRESS Peer Review of Electronic Search Strategies: 2015 Guideline Statement. *J Clin Epidemiol*. 2016;75:40-46. doi:10.1016/j.jclinepi.2016.01.021

22. Sterne JAC, Savović J, Page MJ, et al. RoB 2: a revised tool for assessing risk of bias in randomised trials. *BMJ*. Published online August 28, 2019:l4898. doi:10.1136/bmj.l4898

23. Liu X, Cruz Rivera S, Moher D, et al. Reporting guidelines for clinical trial reports for interventions involving artificial intelligence: the CONSORT-AI extension. *Nat Med*. 2020;26(9):1364-1374. doi:10.1038/s41591-020-1034-x

24. NOT-OD-20-031: Notice of NIH's Interest in Diversity. Accessed February 23, 2022. https://grants.nih.gov/grants/guide/notice-files/NOT-OD-20-031.html

25. Hopewell S, Boutron I, Altman DG, Ravaud P. Incorporation of assessments of risk of bias of primary studies in systematic reviews of randomised trials: a cross-sectional study. *BMJ Open*. 2013;3(8):e003342. doi:10.1136/bmjopen-2013-003342

26. Page MJ, McKenzie JE, Bossuyt PM, et al. The PRISMA 2020 statement: an updated guideline for reporting systematic reviews. *BMJ*. Published online March 29, 2021:n71. doi:10.1136/bmj.n71

27. Kim DW, Jang HY, Kim KW, Shin Y, Park SH. Design Characteristics of Studies Reporting the Performance of Artificial Intelligence Algorithms for Diagnostic Analysis of Medical Images: Results from Recently Published Papers. *Korean J Radiol*. 2019;20(3):405. doi:10.3348/kjr.2019.0025

28. Ben-Israel D, Jacobs WB, Casha S, et al. The impact of machine learning on patient care: A systematic review. *Artif Intell Med*. 2020;103:101785. doi:10.1016/j.artmed.2019.101785

29. FDA. Artificial Intelligence and Machine Learning (AI/ML)-Enabled Medical Devices. FDA. Published September 22, 2021. Accessed March 7, 2022. https://www.fda.gov/medical-devices/software-medical-device-samd/artificial-intelligence-and-machine-learning-aiml-enabled-medical-devices

30. FDA. Software as a Medical Device (SaMD). FDA. Published September 9, 2020. Accessed August 3, 2022. https://www.fda.gov/medical-devices/digital-health-center-excellence/software-medical-device-samd

31. Zhou Q, Chen Z hang, Cao Y heng, Peng S. Clinical impact and quality of randomized controlled trials involving interventions evaluating artificial intelligence prediction tools: a systematic review. *Npj Digit Med*. 2021;4(1):1-12. doi:10.1038/s41746-021-00524-2

32. Nagendran M, Chen Y, Lovejoy CA, et al. Artificial intelligence versus clinicians: systematic review of design, reporting standards, and claims of deep learning studies. *BMJ*. Published online March 25, 2020:m689. doi:10.1136/bmj.m689

33. Ma MA, Gutiérrez DE, Frausto JM, Al-Delaimy WK. Minority Representation in Clinical Trials in the United States. *Mayo Clin Proc*. 2021;96(1):264-266. doi:10.1016/j.mayocp.2020.10.027

34. Hoel AW, Kayssi A, Brahmanandam S, Belkin M, Conte MS, Nguyen LL. Under-representation of women and ethnic minorities in vascular surgery randomized controlled trials. *J Vasc Surg*. 2009;50(2):349-354. doi:10.1016/j.jvs.2009.01.012

35. Center for Devices and Radiological Health. Artificial Intelligence and Machine Learning in Software as a Medical Device. *FDA*. Published online September 22, 2021. Accessed February 23, 2022. https://www.fda.gov/medical-devices/software-medical-device-samd/artificial-intelligence-and-machine-learning-software-medical-device

36. Pavel AM, Rennie JM, de Vries LS, et al. A machine-learning algorithm for neonatal seizure recognition: a multicentre, randomised, controlled trial. *Lancet Child Adolesc Health*. 2020;4(10):740-749. doi:10.1016/S2352-4642(20)30239-X

37. Wang P, Liu P, Glissen Brown JR, et al. Lower Adenoma Miss Rate of Computer-Aided Detection-Assisted Colonoscopy vs Routine White-Light Colonoscopy in a Prospective Tandem Study. *Gastroenterology*. 2020;159(4):1252-1261.e5. doi:10.1053/j.gastro.2020.06.023

38. Caparros-Gonzalez RA, de la Torre-Luque A, Diaz-Piedra C, Vico FJ, Buela-Casal G. Listening to Relaxing Music Improves Physiological Responses in Premature Infants: A Randomized Controlled Trial. *Adv Neonatal Care*. 2018;18(1):58-69. doi:10.1097/ANC.0000000000000448

39. Nimri R, Battelino T, Laffel LM, et al. Insulin dose optimization using an automated artificial intelligence-based decision support system in youths with type 1 diabetes. *Nat Med*. 2020;26(9):1380-1384. doi:10.1038/s41591-020-1045-7

40. Vennalaganti PR, Kaul V, Wang KK, et al. Increased detection of Barrett's esophagus–associated neoplasia using wide-area trans-epithelial sampling: a multicenter, prospective, randomized trial. *Gastrointest Endosc*. 2018;87(2):348-355. doi:10.1016/j.gie.2017.07.039

41. Voss C, Schwartz J, Daniels J, et al. Effect of Wearable Digital Intervention for Improving Socialization in Children With Autism Spectrum Disorder: A Randomized Clinical Trial. *JAMA Pediatr*. 2019;173(5):446. doi:10.1001/jamapediatrics.2019.0285

42. Manz CR, Parikh RB, Small DS, et al. Effect of Integrating Machine Learning Mortality Estimates With Behavioral Nudges to Clinicians on Serious Illness Conversations Among Patients With Cancer: A Stepped-Wedge Cluster Randomized Clinical Trial. *JAMA Oncol*. 2020;6(12):e204759. doi:10.1001/jamaoncol.2020.4759

43. Persell SD, Peprah YA, Lipiszko D, et al. Effect of Home Blood Pressure Monitoring via a Smartphone Hypertension Coaching Application or Tracking Application on Adults With Uncontrolled Hypertension: A Randomized Clinical Trial. *JAMA Netw Open*. 2020;3(3):e200255. doi:10.1001/jamanetworkopen.2020.0255

44. Repici A, Badalamenti M, Maselli R, et al. Efficacy of Real-Time Computer-Aided Detection of Colorectal Neoplasia in a Randomized Trial. *Gastroenterology*. 2020;159(2):512-520.e7. doi:10.1053/j.gastro.2020.04.062

45. Wijnberge M, Geerts BF, Hol L, et al. Effect of a Machine Learning–Derived Early Warning System for Intraoperative Hypotension vs Standard Care on Depth and Duration of Intraoperative Hypotension During Elective Noncardiac Surgery: The HYPE Randomized Clinical Trial. *JAMA*. 2020;323(11):1052. doi:10.1001/jama.2020.0592

© 2022 Plana D et al. *JAMA Network Open*.

46. Shimabukuro DW, Barton CW, Feldman MD, Mataraso SJ, Das R. Effect of a machine learning-based severe sepsis prediction algorithm on patient survival and hospital length of stay: a randomised clinical trial. *BMJ Open Respir Res*. 2017;4(1):e000234. doi:10.1136/bmjresp-2017-000234

47. Wang P, Liu X, Berzin TM, et al. Effect of a deep-learning computer-aided detection system on adenoma detection during colonoscopy (CADe-DB trial): a double-blind randomised study. *Lancet Gastroenterol Hepatol*. 2020;5(4):343-351. doi:10.1016/S2468-1253(19)30411-X

48. Gong D, Wu L, Zhang J, et al. Detection of colorectal adenomas with a real-time computer-aided system (ENDOANGEL): a randomised controlled study. *Lancet Gastroenterol Hepatol*. 2020;5(4):352-361. doi:10.1016/S2468-1253(19)30413-3

49. Lin H, Li R, Liu Z, et al. Diagnostic Efficacy and Therapeutic Decision-making Capacity of an Artificial Intelligence Platform for Childhood Cataracts in Eye Clinics: A Multicentre Randomized Controlled Trial. *EClinicalMedicine*. 2019;9:52-59. doi:10.1016/j.eclinm.2019.03.001

50. Rabbi M, Pfammatter A, Zhang M, Spring B, Choudhury T. Automated Personalized Feedback for Physical Activity and Dietary Behavior Change With Mobile Phones: A Randomized Controlled Trial on Adults. *JMIR MHealth UHealth*. 2015;3(2):e42. doi:10.2196/mhealth.4160

51. Auloge P, Cazzato RL, Ramamurthy N, et al. Augmented reality and artificial intelligence-based navigation during percutaneous vertebroplasty: a pilot randomised clinical trial. *Eur Spine J*. 2020;29(7):1580-1589. doi:10.1007/s00586-019-06054-6

52. Avari P, Leal Y, Herrero P, et al. Safety and Feasibility of the PEPPER Adaptive Bolus Advisor and Safety System: A Randomized Control Study. *Diabetes Technol Ther*. 2021;23(3):175-186. doi:10.1089/dia.2020.0301

53. Wang P, Berzin TM, Glissen Brown JR, et al. Real-time automatic detection system increases colonoscopic polyp and adenoma detection rates: a prospective randomised controlled study. *Gut*. 2019;68(10):1813-1819. doi:10.1136/gutjnl-2018-317500

54. Forman EM, Goldstein SP, Crochiere RJ, et al. Randomized controlled trial of OnTrack, a just-in-time adaptive intervention designed to enhance weight loss. *Transl Behav Med*. 2019;9(6):989-1001. doi:10.1093/tbm/ibz137

55. Wu L, Zhang J, Zhou W, et al. Randomised controlled trial of WISENSE, a real-time quality improving system for monitoring blind spots during esophagogastroduodenoscopy. *Gut*. 2019;68(12):2161-2169. doi:10.1136/gutjnl-2018-317366

56. El Solh A, Akinnusi M, Patel A, Bhat A, TenBrock R. Predicting optimal CPAP by neural network reduces titration failure: a randomized study. *Sleep Breath*. 2009;13(4):325-330. doi:10.1007/s11325-009-0247-5

57. Luštrek M, Bohanec M, Cavero Barca C, et al. A Personal Health System for Self-Management of Congestive Heart Failure (HeartMan): Development, Technical Evaluation, and Proof-of-Concept Randomized Controlled Trial. *JMIR Med Inform*. 2021;9(3):e24501. doi:10.2196/24501

58. Chen J, Gao Y. The Role of Deep Learning-Based Echocardiography in the Diagnosis and Evaluation of the Effects of Routine Anti-Heart-Failure Western Medicines in Elderly Patients with Acute Left Heart Failure. Singh D, ed. *J Healthc Eng*. 2021;2021:1-9. doi:10.1155/2021/4845792

59. Seol HY, Shrestha P, Muth JF, et al. Artificial intelligence-assisted clinical decision support for childhood asthma management: A randomized clinical trial. Leroyer C, ed. *PLOS ONE*. 2021;16(8):e0255261. doi:10.1371/journal.pone.0255261

© 2022 Plana D et al. *JAMA Network Open*.

60. Repici A, Spadaccini M, Antonelli G, et al. Artificial intelligence and colonoscopy experience: lessons from two randomised trials. *Gut*. 2022;71(4):757-765. doi:10.1136/gutjnl-2021-324471

61. Kamba S, Tamai N, Saitoh I, et al. Reducing adenoma miss rate of colonoscopy assisted by artificial intelligence: a multicenter randomized controlled trial. *J Gastroenterol*. 2021;56(8):746-757. doi:10.1007/s00535-021-01808-w

62. Liu P, Wang P, Glissen Brown JR, et al. The single-monitor trial: an embedded CADe system increased adenoma detection during colonoscopy: a prospective randomized study. *Ther Adv Gastroenterol*. 2020;13:175628482097916. doi:10.1177/1756284820979165

63. Blomberg SN, Christensen HC, Lippert F, et al. Effect of Machine Learning on Dispatcher Recognition of Out-of-Hospital Cardiac Arrest During Calls to Emergency Medical Services: A Randomized Clinical Trial. *JAMA Netw Open*. 2021;4(1):e2032320. doi:10.1001/jamanetworkopen.2020.32320

64. Xu L, He X, Zhou J, et al. Artificial intelligence-assisted colonoscopy: A prospective, multicenter, randomized controlled trial of polyp detection. *Cancer Med*. 2021;10(20):7184-7193. doi:10.1002/cam4.4261

65. Jayakumar P, Moore MG, Furlough KA, et al. Comparison of an Artificial Intelligence–Enabled Patient Decision Aid vs Educational Material on Decision Quality, Shared Decision-Making, Patient Experience, and Functional Outcomes in Adults With Knee Osteoarthritis: A Randomized Clinical Trial. *JAMA Netw Open*. 2021;4(2):e2037107. doi:10.1001/jamanetworkopen.2020.37107

66. Wu L, He X, Liu M, et al. Evaluation of the effects of an artificial intelligence system on endoscopy quality and preliminary testing of its performance in detecting early gastric cancer: a randomized controlled trial. *Endoscopy*. 2021;53(12):1199-1207. doi:10.1055/a-1350-5583

67. Sandal LF, Bach K, Øverås CK, et al. Effectiveness of App-Delivered, Tailored Self-management Support for Adults With Lower Back Pain–Related Disability: A SELF BACK Randomized Clinical Trial. *JAMA Intern Med*. 2021;181(10):1288. doi:10.1001/jamainternmed.2021.4097

68. Noor N, Vignarajan C, Malhotra N, Vanamail P. Three-Dimensional Automated Volume Calculation (Sonography-Based Automated Volume Count) versus Two-Dimensional Manual Ultrasonography for Follicular Tracking and Oocyte Retrieval in Women Undergoing in vitro Fertilization-Embryo Transfer: A Randomized Controlled Trial. *J Hum Reprod Sci*. 2020;13(4):296. doi:10.4103/jhrs.JHRS_91_20

69. Yao X, Rushlow DR, Inselman JW, et al. Artificial intelligence–enabled electrocardiograms for identification of patients with low ejection fraction: a pragmatic, randomized clinical trial. *Nat Med*. 2021;27(5):815-819. doi:10.1038/s41591-021-01335-4

70. Wu L, Shang R, Sharma P, et al. Effect of a deep learning-based system on the miss rate of gastric neoplasms during upper gastrointestinal endoscopy: a single-centre, tandem, randomised controlled trial. *Lancet Gastroenterol Hepatol*. 2021;6(9):700-708. doi:10.1016/S2468-1253(21)00216-8

71. Strömblad CT, Baxter-King RG, Meisami A, et al. Effect of a Predictive Model on Planned Surgical Duration Accuracy, Patient Wait Time, and Use of Presurgical Resources: A Randomized Clinical Trial. *JAMA Surg*. 2021;156(4):315. doi:10.1001/jamasurg.2020.6361

72. Eng DK, Khandwala NB, Long J, et al. Artificial Intelligence Algorithm Improves Radiologist Performance in Skeletal Age Assessment: A Prospective Multicenter Randomized Controlled Trial. *Radiology*. 2021;301(3):692-699. doi:10.1148/radiol.2021204021

73. Glissen Brown JR, Mansour NM, Wang P, et al. Deep Learning Computer-aided Polyp Detection Reduces Adenoma Miss Rate: A United States Multi-center Randomized Tandem Colonoscopy Study (CADeT-CS Trial). *Clin Gastroenterol Hepatol*. 2022;20(7):1499-1507.e4. doi:10.1016/j.cgh.2021.09.009

© 2022 Plana D et al. *JAMA Network Open*.

74. Meijer F, Honing M, Roor T, et al. Reduced postoperative pain using Nociception Level-guided fentanyl dosing during sevoflurane anaesthesia: a randomised controlled trial. *Br J Anaesth*. 2020;125(6):1070-1078. doi:10.1016/j.bja.2020.07.057

75. Liu WN, Zhang YY, Bian XQ, et al. Study on detection rate of polyps and adenomas in artificial-intelligence-aided colonoscopy. *Saudi J Gastroenterol*. 2020;26(1):13. doi:10.4103/sjg.SJG_377_19

76. Su JR, Li Z, Shao XJ, et al. Impact of a real-time automatic quality control system on colorectal polyp and adenoma detection: a prospective randomized controlled study (with videos). *Gastrointest Endosc*. 2020;91(2):415-424.e4. doi:10.1016/j.gie.2019.08.026

© 2022 Plana D et al. *JAMA Network Open*.
